# Reasons for Suicide During the COVID-19 Pandemic in Japan

**DOI:** 10.1001/jamanetworkopen.2021.45870

**Published:** 2022-01-31

**Authors:** Masahide Koda, Nahoko Harada, Akifumi Eguchi, Shuhei Nomura, Yasushi Ishida

**Affiliations:** 1 Department of Psychiatry, Faculty of Medicine, University of Miyazaki, Miyazaki, Japan; 2 Department of Psychiatric and Mental Health Nursing, School of Nursing, University of Miyazaki, Miyazaki, Japan; 3 Department of Sustainable Health Science, Center for Preventive Medical Sciences, Chiba University, Chiba, Japan; 4 Department of Health Policy and Management, School of Medicine, Keio University, Tokyo, Japan; 5 Department of Global Health Policy, Graduate School of Medicine, The University of Tokyo, Tokyo, Japan; 6 Tokyo Foundation for Policy Research, Tokyo, Japan

## Abstract

**Importance:**

Although the suicide rate in Japan increased during the COVID-19 pandemic, the reasons for suicide have yet to be comprehensively investigated.

**Objective:**

To assess which reasons for suicide had rates that exceeded the expected number of suicide deaths for that reason during the COVID-19 pandemic.

**Design, Setting, and Participants:**

This national, population-based cross-sectional study of data on suicides gathered by the Ministry of Health, Labor, and Welfare from January 2020 to May 2021 used a times-series analysis on the numbers of reason-identified suicides. Data of decedents were recorded by the National Police Agency and compiled by the Ministry of Health, Labor, and Welfare.

**Exposure:**

For category analysis, we compared data from January 2020 to May 2021 with data from December 2014 to June 2020. For subcategory analysis, data from January 2020 to May 2021 were compared with data from January 2019 to June 2020.

**Main Outcomes and Measures:**

The main outcome was the monthly excess suicide rate, ie, the difference between the observed number of monthly suicide deaths and the upper bound of the 1-sided 95% CI for the expected number of suicide deaths in that month. Reasons for suicide were categorized into family, health, economy, work, relationships, school, and others, which were further divided into 52 subcategories. A quasi-Poisson regression model was used to estimate the expected number of monthly suicides. Individual regression models were used for each of the 7 categories, 52 subcategories, men, women, and both genders.

**Results:**

From the 29 938 suicides (9984 [33.3%] women; 1093 [3.7%] aged <20 years; 3147 [10.5%] aged >80 years), there were 21 027 reason-identified suicides (7415 [35.3%] women). For both genders, all categories indicated monthly excess suicide rates, except for school in men. October 2020 had the highest excess suicide rates for all cases (observed, 1577; upper bound of 95% CI for expected number of suicides, 1254; 25.8% greater). In men, the highest monthly excess suicide rate was 24.3% for the other category in August 2020 (observed, 87; upper bound of 95% CI for expected number, 70); in women, it was 85.7% for school in August 2020 (observed, 26; upper bound of 95% CI for expected number, 14).

**Conclusions and Relevance:**

In this study, observed suicides corresponding to all 7 categories of reasons exceeded the monthly estimates (based on data from before or during the COVID-19 pandemic), except for school-related reasons in men. This study can be used as a basis for developing intervention programs for suicide prevention.

## Key Points

**Question:**

Is the COVID-19 pandemic associated with changes in the reasons for suicide in Japan?

**Findings:**

In this cross-sectional study of 21 027 reason-identified suicides, all categories of reasons for suicide had monthly excess suicide rates during the COVID-19 pandemic, except for school in men. There were gender differences in subcategories.

**Meaning:**

The findings of this study could help to develop gender-specific suicide prevention interventions and programs.

## Teaser

This cross-sectional study assesses whether the COVID-19 pandemic was associated with changes in reasons for suicide among individuals in Japan.

## Introduction

According to the World Health Organization (WHO),^1^ suicide is a critical public health concern. Furthermore, research has shown that infectious diseases (eg, COVID-19) adversely affect mental health.^2^ Nonetheless, suicide rates in different countries seem to have been constant since the COVID-19 pandemic onset^3,4,5,6^; in contrast, Japan’s statistics have indicated an increasing trend.^7,8^ Worldwide, suicide rates tend to be higher in men than women.^1,9,10^ However, from July to October 2020 (ie, the second wave of the COVID-19 pandemic), the rate of increase in suicide rates was higher among Japanese women than men.^11,12^ Therefore, the COVID-19 pandemic may be associated with changes in the reasons for suicide among the Japanese population.

Generally, the reasons for suicide tend to be multifactoral,^13^ and the following have been related to suicide: depression, environment, economic status, gender, and society^14^; sociocultural behavioral norms (ie, while men are less likely to engage in help-seeking behaviors owing to masculinism, women are more likely to do so^10,15,16,17^); and media reporting, as careless suicide reporting may trigger the Werther effect, ie, copycat suicides.^18,19^ Additionally, the following have been associated with poorer mental health during the COVID-19 pandemic: interpersonal distress, parenting challenges, marital discord, alienation, and loneliness.^20,21,22,23^

In Japan, suicide was a major public health issue even before the COVID-19 pandemic. In 1998, the annual number of suicide cases exceeded 32 000, and similar numbers were recorded until 2010.^24^ In Japanese culture, men are supposed to be family breadwinners and women, the caregivers and homemakers.^25^ Accordingly, the most identified reason for suicide among employed Japanese men has been economic problems.^26^ A strong social stigma also prevents suicide from being thoroughly investigated in Japan.^24^ Amid this reality, the WHO asked the Japanese government to devise countermeasures to curb national suicide rates.^24^ In 2006, the Japanese government passed the Basic Act on Suicide Prevention, aimed at preventing suicide.^27^ As a result, suicide numbers dropped to less than 30 000 in 2012; suicide ratios were the highest in 2013 (27.0 per 100 000 people) and the lowest in 2019 (16.0 per 100 000 people).^24^

To prevent the spread of COVID-19, the Japanese government issued various restrictive measures (eg, limited public transportation) that could have induced psychological distress and job loss nationwide^28,29,30^; these negative consequences of the battle against COVID-19 may have affected men and women differently. During school closures because of COVID-19, Japanese mothers had worsened mental health, while fathers did not.^30^ Furthermore, depression has been associated with the role of family caregiver, and being a woman was a risk factor for suicidal behaviors during the pandemic.^31,32,33^ Amid the COVID-19 pandemic, risk factors for depression, anxiety, and physical health were disproportionally higher in Japanese women than in men.^33,34^ Additionally, ever since the onset of the COVID-19 pandemic, there has been an increase in suicide rates among women, a phenomenon that has also been observed internationally.^35,36^

Considering that the period of increase in suicide rates during the COVID-19 pandemic has been the longest compared with that of all other large-scale natural disasters,^37^ it is necessary to implement optimal suicide prevention measures in Japan. Furthermore, although several studies have highlighted that the COVID-19 pandemic has heightened the risk of poor mental health, few have used national-level data to examine suicide in this period. A systematic review has warned about the low quality of the design and sampling of extant studies,^38^ and studies that used national-level data to examine mental health burden owing to the COVID-19 pandemic^39,40,41,42^ have not directly explored the reasons for suicide. This study aimed to assess which reasons for suicide had higher monthly numbers during the COVID-19 pandemic than the estimated number of suicide deaths for that month.

## Methods

For this national-level cross-sectional time-series analysis, we extracted publicly available data from government sources on the number of suicide deaths for which the reason was known (ie, reason-identified suicide). Although we used only publicly available data, we obtained approval from the Medical Ethics Review Committee of the University of Miyazaki and adhered to the Strengthening the Reporting of Observational Studies in Epidemiology (STROBE) and the International COVID-19 Suicide Prevention Research Collaboration reporting guidelines.^43^ Informed consent was waived because this study used secondary data.

### National Suicide Statistics

In Japan, statistics on suicide are compiled by the Ministry of Health, Labor, and Welfare (MHLW), recorded by the National Police Agency (NPA), and often used in related studies.^8,10,11^ In Japan, only doctors can prepare death certificates, and the Medical Practitioners Law stipulates that an abnormal death must be reported to the NPA within 24 hours.^44^ Through criminal investigation, the NPA must examine all corpses with abnormal causes of death and determine the cause of death; they conduct physiological tests, examine suicide notes and emails, conduct interviews with family members, and assess documents (eg, doctor’s notes, medical certificates, loans).^45,46,47^ The national record system requires the NPA to register 1 to 3 reasons for suicide, an action aimed at helping improve suicide prevention strategies.^48^ After accumulating data, the number of suicide deaths and their reasons are published by biological sex and age group (10-year increments), except for those younger than 19 or older than 80 years. To ensure confidentiality, the data set does not contain exact age, gender, or geographical information.

According to the Suicide Countermeasures Basic Law established in 2007 by the Japanese government,^48^ there are 7 categories (and 52 subcategories) of reasons for suicide: family, health, economy, work, relationship, school, others (eg, copycat suicide), and unknown. We excluded the unknown category because the NPA updates statistics when the suicide was identified.

There are 52 subcategories, as follows: for family, there are parent-child problems, marital discord, other family discords, death of a family member, pessimism about the future of the family, abuse from family, child-rearing problems, abuse, caregiving fatigue, and others. For health, there are physical illness, depression, schizophrenia, alcoholism, drug and substance abuse, other mental disorders, physical disability, and others. For economy, there are bankruptcy, business slump, unemployment, job failure, poverty, multiple debts, joint guarantee, other debts, debt collection trouble, suicide for insurance, and others. For work, there are work failure, workplace relationships, work environment changes, work fatigue, and others. For relationships, there are marriage, heartbreak, infidelity, other relationship distress, and others. For school, there are admission, academic path, academic failure, issues with teachers, bullying, schoolmate trouble, and others. For others, there are discovery of a crime, victim of crime, copycat suicide, loneliness, neighborhood trouble, and others.

### Statistical Analysis

Although monthly data on the categories are available from January 2010, data on the subcategories are available only from January 2019. Therefore, for the 7 categories, we used monthly data from January 2010 to May 2021 (latest data available as of July 2021); for the subcategories, and to analyze data since the first COVID-19 case in Japan (ie, January 2020), we used monthly data from January 2019 to May 2021.

We used quasi-Poisson regression to estimate the expected number of monthly suicide deaths. To assess the parameters of the quasi-Poisson regression, we used the Farrington algorithm.^49,50^ We constructed separate models for men, women, both genders combined, all cases, each category, and each subcategory. For the categories, we assumed that the number of suicide deaths in a month during the COVID-19 pandemic would remain similar to that recorded in the past 5 years for that given month and the months immediately before and after; then, we estimated the extent to which the observed number of monthly suicide deaths differed from this assumption.^51,52^ For the subcategories (ie, available from 2019), we used data from 1 month before and 1 year after the corresponding month.

In each estimation, we incorporated a trend term (ie, data trends over time, such as a constant increase or decrease) and seasonality (ie, a regular pattern of changes) into the model. The monthly excess suicide rates were calculated by the formula: observed suicides minus the upper bound of the 95% CI of the expected number of suicides, divided by the upper bound. The results were interpreted as the suicide burden associated with the COVID-19 pandemic. For this 1-sided analysis, we defined statistical significance at 5%. We used R version 4.1.0 (R Project for Statistical Computing) for all analyses and graphical representations and the surveillance package for the Farrington algorithm.^52^

## Results

### Overall Observations

In the 17 months between January 2020 and May 2021, 29 938 people died of suicide (9984 [33.3%] women; 1093 [3.7%] aged <20 years; 3147 [10.5%] aged >80 years) (Table 1; eTable 1 in the Supplement). In total, there were 21 027 reason-identified suicides (70.2%; 7415 [35.3%] women). More than 70% of suicides without an identified reason were among men (6342 [71.2%]). A χ^2^ analysis indicated a significant difference between men and women in the percentage of suicides with a known or unknown reason (χ^2^_1_ = 116.3; *P* < .001).

**Table 1.** Suicides Overall and by Gender From January 2020 to May 2021 by Age Group

Group	All ages, No.	Individuals by age group, No. (%)								
		≤19 y	20-29 y	30-39 y	40-49 y	50-59 y	60-69 y	70-79 y	≥80 y	Unknown
**Total**										
Both genders	29 938	1093 (3.7)	3664 (12.2)	3697 (12.3)	5106 (17.1)	4922 (16.4)	3954 (13.2)	4265 (14.2)	3147 (10.5)	90 (0.3)
Men	19 954	657 (3.3)	2427 (12.2)	2634 (13.2)	3527 (17.7)	3404 (17.1)	2641 (13.2)	2675 (13.4)	1912 (9.6)	77 (0.4)
Women	9984	436 (4.4)	1237 (12.4)	1063 (10.6)	1579 (15.8)	1518 (15.2)	1313 (13.2)	1590 (15.9)	1235 (12.4)	13 (0.1)
**Reason-identified suicides**										
Both genders	21 027	699 (3.3)	2520 (12.0)	2641 (12.6)	3561 (16.9)	3465 (16.5)	2856 (13.6)	3045 (14.5)	2230 (10.6)	10 (<0.1)
Men	13 612	388 (2.9)	1624 (11.9)	1820 (13.4)	2392 (17.6)	2325 (17.1)	1878 (13.8)	1850 (13.6)	1327 (9.7)	8 (0.1)
Women	7415	311 (4.2)	896 (12.1)	821 (11.1)	1169 (15.8)	1140 (15.4)	978 (13.2)	1195 (16.1)	903 (12.2)	2 (<0.1)
**Reasons**										
Family										
Both genders	4382	191 (4.4)	415 (9.5)	594 (13.6)	803 (18.3)	750 (17.1)	547 (12.5)	606 (13.8)	476 (10.9)	0
Men	2567	104 (4.1)	246 (9.6)	364 (14.2)	497 (19.4)	419 (16.3)	324 (12.6)	337 (13.1)	276 (10.8)	0
Women	1815	87 (4.8)	169 (9.3)	230 (12.7)	306 (16.9)	331 (18.2)	223 (12.3)	269 (14.8)	200 (11)	0
Health										
Both genders	13 940	237 (1.7)	1110 (8.0)	1376 (9.9)	2130 (15.3)	2239 (16.1)	2172 (15.6)	2673 (19.2)	1997 (14.3)	6 (<0.1)
Men	7796	98 (1.3)	539 (6.9)	755 (9.7)	1167 (15.0)	1263 (16.2)	1256 (16.1)	1534 (19.7)	1179 (15.1)	5 (0.1)
Women	6144	139 (2.3)	571 (9.3)	621 (10.1)	963 (15.7)	976 (15.9)	916 (14.9)	1139 (18.5)	818 (13.3)	1 (<0.1)
Economy										
Both genders	4578	20 (0.4)	582 (12.7)	713 (15.6)	977 (21.3)	1105 (24.1)	765 (16.7)	336 (7.3)	76 (1.7)	4 (0.1)
Men	3964	14 (0.4)	499 (12.6)	622 (15.7)	861 (21.7)	960 (24.2)	685 (17.3)	272 (6.9)	48 (1.2)	3 (0.1)
Women	614	6 (1.0)	83 (13.5)	91 (14.8)	116 (18.9)	145 (23.6)	80 (13.0)	64 (10.4)	28 (4.6)	1 (0.2)
Work										
Both genders	2690	47 (1.7)	576 (21.4)	541 (20.1)	689 (25.6)	576 (21.4)	202 (7.5)	49 (1.8)	10 (0.4)	0
Men	2241	38 (1.7)	430 (19.2)	445 (19.9)	585 (26.1)	509 (22.7)	180 (8.0)	45 (2.0)	9 (0.4)	0
Women	449	9 (2.0)	146 (32.5)	96 (21.4)	104 (23.2)	67 (14.9)	22 (4.9)	4 (0.9)	1 (0.2)	0
Relationships										
Both genders	1112	83 (7.5)	343 (30.8)	320 (28.8)	214 (19.2)	103 (9.3)	28 (2.5)	18 (1.6)	3 (0.3)	0
Men	654	47 (7.2)	171 (26.1)	205 (31.3)	124 (19.0)	67 (10.2)	25 (3.8)	13 (2)	2 (0.3)	0
Women	458	36 (7.9)	172 (37.6)	115 (25.1)	90 (19.7)	36 (7.9)	3 (0.7)	5 (1.1)	1 (0.2)	0
School										
Both genders	552	303 (54.9)	235 (42.6)	13 (2.4)	1 (0.2)	0	0	0	0	0
Men	353	171 (48.4)	171 (48.4)	11 (3.1)	0	0	0	0	0	0
Women	199	132 (66.3)	64 (32.2)	2 (1.0)	1 (0.5)	0	0	0	0	0
Others										
Both genders	1704	82 (4.8)	248 (14.6)	205 (12.0)	268 (15.7)	229 (13.4)	205 (12.0)	234 (13.7)	233 (13.7)	0
Men	1151	47 (4.1)	168 (14.6)	148 (12.9)	195 (16.9)	173 (15.0)	138 (12.0)	158 (13.7)	124 (10.8)	0
Women	553	35 (6.3)	80 (14.5)	57 (10.3)	73 (13.2)	56 (10.1)	67 (12.1)	76 (13.7)	109 (19.7)	0
**Unknown reason**										
Both genders	8911	394 (4.4)	1144 (12.8)	1056 (11.9)	1545 (17.3)	1457 (16.4)	1098 (12.3)	1220 (13.7)	917 (10.3)	80 (0.9)
Men	6342	269 (4.2)	803 (12.7)	814 (12.8)	1135 (17.9)	1079 (17.0)	763 (12.0)	825 (13.0)	585 (9.2)	69 (1.1)
Women	2569	125 (4.9)	341 (13.3)	242 (9.4)	410 (16.0)	378 (14.7)	335 (13.0)	395 (15.4)	332 (12.9)	11 (0.4)

In the quasi-Poisson regression model of the total number of reason-identified suicides, there were 5 months in which the number of deaths exceeded the assumption (ie, July to November 2020) (Figure). In men, there were 2 months with excess suicide rates (October and November 2020); in women, there were 7, with 6 being consecutive months (July to December 2020 and March 2021). By month, October 2020 had the highest excess suicide rates for all cases (observed, 1577; upper bound of 95% CI for expected number of suicides, 1254; 25.8% greater) (Table 2). Among the 7 categories, the highest excess suicide rate for all cases was related to health in October 2020 (observed, 1099; upper bound of 95% CI for expected number, 831; 32.3% greater) (Table 3; eFigure 1 in the Supplement). In women, we observed excess suicide rates for 5 consecutive months in family, health, and work and for 6 consecutive months in other reason.

**Figure.**
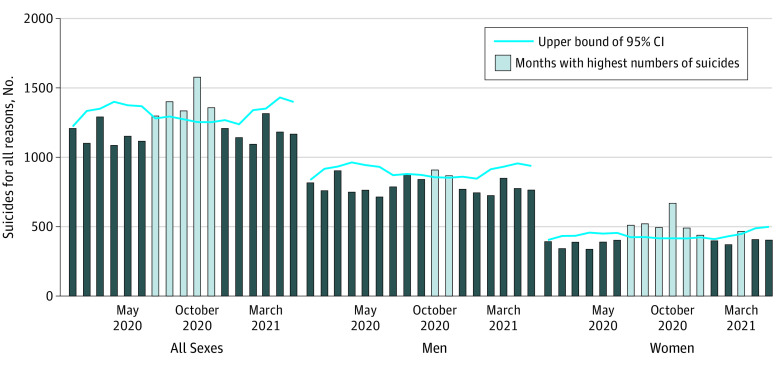
Suicides Overall and by Gender From January 2020 to May 2021 Information about how the expected number of suicides per month appears in the Methods section.

**Table 2.** Expected and Observed Number of Monthly Suicides and Percentage Change From January 2020 to May 2021 Overall and by Gender

Month and year	Both genders			Men			Women		
	Expected (upper bound), No.	Observed, No.	Change, %	Expected (upper bound), No.	Observed, No.	Change, %	Expected (upper bound), No.	Observed, No.	Change, %
Jan 2020	1101 (1222)	1208	−1.1	746 (837)	816	−2.5	360 (404)	392	−3.0
Feb 2020	1177 (1334)	1101	−17.5	802 (917)	759	−17.2	381 (433)	342	−21.0
Mar 2020	1194 (1350)	1291	−4.4	816 (933)	903	−3.2	379 (434)	388	−10.6
Apr 2020	1265 (1400)	1086	−22.4	863 (963)	749	−22.2	409 (457)	337	−26.3
May 2020	1225 (1375)	1152	−16.2	833 (944)	763	−19.2	400 (450)	389	−13.6
Jun 2020	1220 (1368)	1116	−18.4	820 (931)	714	−23.3	399 (455)	402	−11.6
Jul 2020	1160 (1280)	1297	1.3^a^	781 (871)	787	−9.6	378 (424)	510	20.3^a^
Aug 2020	1156 (1294)	1400	8.2^a^	780 (880)	880	0.0	376 (425)	520	22.4^a^
Sep 2020	1137 (1274)	1334	4.7^a^	771 (873)	840	−3.8	366 (416)	494	18.8^a^
Oct 2020	1139 (1254)	1577	25.8^a^	768 (856)	908	6.1^a^	371 (416)	669	60.8^a^
Nov 2020	1115 (1253)	1357	8.3^a^	750 (853)	867	1.6^a^	365 (415)	490	18.1^a^
Dec 2020	1131 (1268)	1208	−4.7	758 (860)	770	−10.5	373 (423)	438	3.5^a^
Jan 2021	1118 (1238)	1142	−7.8	755 (846)	744	−12.1	363 (410)	398	−2.9
Feb 2021	1200 (1340)	1094	−18.4	812 (914)	724	−20.8	380 (431)	370	−14.2
Mar 2021	1211 (1351)	1315	−2.7	824 (932)	849	−8.9	392 (447)	466	4.3^a^
Apr 2021	1292 (1431)	1182	−17.4	859 (956)	775	−18.9	427 (488)	407	−16.6
May 2021	1247 (1399)	1167	−16.6	828 (938)	764	−18.6	430 (499)	403	−19.2

^a^ A month with the observed number of suicides exceeding the 95% upper bound of the expected number of suicides for that month. Percentage change was defined as the difference between the observed number of suicides for a month and the 95% upper bound of the expected number of suicides for that month divided by the threshold.

**Table 3.** Expected and Observed Number of Monthly Suicides and Percentage Change by Reasons for Suicide, Overall and by Gender

Month and year	Both genders			Men			Women		
	Expected (upper bound), No.	Observed, No.	Change, %	Expected (upper bound), No.	Observed, No.	Change, %	Expected (upper bound), No.	Observed, No.	Change, %
Family									
Jan 2020	228 (262)	238	−9.2	141 (167)	148	−11.4	88 (108)	90	−16.7
Feb 2020	245 (284)	228	−19.7	152 (180)	142	−21.1	92 (112)	86	−23.2
Mar 2020	249 (288)	302	4.9^a^	152 (181)	195	7.7^a^	97 (118)	107	−9.3
Apr 2020	260 (296)	209	−29.4	159 (186)	139	−25.3	102 (122)	70	−42.6
May 2020	248 (284)	234	−17.6	151 (177)	154	−13.0	97 (118)	80	−32.2
Jun 2020	244 (281)	247	−12.1	146 (173)	144	−16.8	98 (119)	103	−13.4
Jul 2020	239 (271)	244	−10.0	141 (165)	126	−23.6	98 (118)	118	0.0
Aug 2020	242 (276)	268	−2.9	142 (166)	169	1.8^a^	100 (121)	99	−18.2
Sep 2020	236 (270)	291	7.8^a^	139 (164)	163	−0.6	98 (119)	128	7.6^a^
Oct 2020	232 (263)	313	19.0^a^	138 (161)	148	−8.1	94 (114)	165	44.7^a^
Nov 2020	225 (261)	297	13.8^a^	134 (159)	169	6.3^a^	91 (112)	128	14.3^a^
Dec 2020	229 (266)	257	−3.4	139 (166)	139	−16.3	90 (111)	118	6.3^a^
Jan 2021	231 (264)	247	−6.4	142 (167)	127	−24.0	88 (108)	120	11.1^a^
Feb 2021	249 (286)	242	−15.4	154 (180)	135	−25.0	95 (116)	107	−7.8
Mar 2021	254 (292)	271	−7.2	157 (186)	154	−17.2	96 (118)	117	−0.8
Apr 2021	264 (303)	243	−19.8	163 (190)	164	−13.7	101 (124)	79	−36.3
May 2021	251 (289)	251	−13.1	153 (179)	151	−15.6	99 (121)	100	−17.4
Health									
Jan 2020	711 (794)	740	−6.8	421 (479)	424	−11.5	294 (334)	316	−5.4
Feb 2020	740 (843)	697	−17.3	449 (523)	416	−20.5	303 (346)	281	−18.8
Mar 2020	765 (871)	806	−7.5	458 (531)	478	−10.0	307 (356)	328	−7.9
Apr 2020	813 (904)	734	−18.8	493 (560)	421	−24.8	335 (382)	313	−18.1
May 2020	805 (905)	795	−12.2	488 (560)	453	−19.1	329 (374)	342	−8.6
Jun 2020	826 (931)	829	−11.0	490 (560)	472	−15.7	336 (389)	357	−8.2
Jul 2020	767 (853)	958	12.3^a^	467 (528)	523	−0.9	316 (364)	435	19.5^a^
Aug 2020	750 (845)	997	18.0^a^	460 (527)	560	6.3^a^	308 (357)	437	22.4^a^
Sep 2020	750 (848)	892	5.2^a^	450 (515)	475	−7.8	300 (347)	417	20.2^a^
Oct 2020	755 (831)	1099	32.3^a^	442 (500)	522	4.4^a^	313 (360)	577	60.3^a^
Nov 2020	736 (828)	879	6.2^a^	431 (498)	497	−0.2	309 (354)	382	7.9^a^
Dec 2020	742 (838)	769	−8.2	426 (488)	435	−10.9	316 (364)	334	−8.2
Jan 2021	713 (794)	714	−10.1	420 (478)	389	−18.6	299 (345)	325	−5.8
Feb 2021	755 (853)	699	−18.1	448 (513)	405	−21.1	310 (359)	294	−18.1
Mar 2021	782 (880)	795	−9.7	463 (530)	442	−16.6	325 (376)	353	−6.1
Apr 2021	845 (940)	775	−17.6	491 (553)	433	−21.7	364 (423)	342	−19.1
May 2021	852 (968)	762	−21.3	489 (561)	451	−19.6	369 (433)	311	−28.2
Economy									
Jan 2020	242 (292)	321	9.9^a^	212 (257)	284	10.5^a^	32 (43)	37	−14.0
Feb 2020	266 (320)	278	−13.1	233 (282)	254	−9.9	35 (47)	24	−48.9
Mar 2020	267 (325)	328	0.9^a^	245 (302)	291	−3.6	34 (47)	37	−21.3
Apr 2020	298 (357)	275	−23.0	260 (314)	238	−24.2	38 (50)	37	−26.0
May 2020	273 (325)	233	−28.3	239 (287)	203	−29.3	35 (47)	30	−36.2
Jun 2020	266 (322)	182	−43.5	231 (282)	162	−42.6	36 (49)	20	−59.2
Jul 2020	245 (292)	225	−22.9	214 (258)	190	−26.4	32 (43)	35	−18.6
Aug 2020	249 (298)	228	−23.5	217 (260)	193	−25.8	33 (45)	35	−22.2
Sep 2020	249 (302)	287	−5.0	221 (273)	252	−7.7	32 (44)	35	−20.5
Oct 2020	257 (310)	302	−2.6	224 (272)	250	−8.1	34 (45)	52	15.6^a^
Nov 2020	247 (296)	265	−10.5	216 (262)	226	−13.7	34 (47)	39	−17.0
Dec 2020	258 (314)	292	−7.0	226 (280)	248	−11.4	35 (48)	44	−8.3
Jan 2021	263 (321)	274	−14.6	231 (284)	243	−14.4	33 (44)	31	−29.5
Feb 2021	286 (342)	242	−29.2	260 (312)	206	−34.0	35 (49)	36	−26.5
Mar 2021	292 (357)	311	−12.9	256 (314)	269	−14.3	36 (50)	42	−16.0
Apr 2021	301 (362)	271	−25.1	262 (318)	238	−25.2	40 (52)	33	−36.5
May 2021	277 (332)	264	−20.5	241 (292)	217	−25.7	38 (52)	47	−9.6
Work									
Jan 2020	154 (184)	171	−7.1	138 (166)	144	−13.3	16 (23)	27	17.4^a^
Feb 2020	162 (192)	120	−37.5	143 (171)	108	−36.8	18 (26)	12	−53.8
Mar 2020	164 (197)	170	−13.7	145 (174)	148	−14.9	19 (28)	22	−21.4
Apr 2020	174 (205)	132	−35.6	152 (180)	113	−37.2	22 (30)	19	−36.7
May 2020	170 (201)	130	−35.3	148 (177)	111	−37.3	22 (30)	19	−36.7
Jun 2020	165 (197)	121	−38.6	144 (173)	100	−42.2	21 (31)	21	−32.3
Jul 2020	155 (183)	171	−6.6	135 (162)	132	−18.5	20 (28)	39	39.3^a^
Aug 2020	153 (183)	161	−12.0	134 (161)	131	−18.6	20 (29)	30	3.4^a^
Sep 2020	149 (180)	164	−8.9	129 (157)	126	−19.7	20 (30)	38	26.7^a^
Oct 2020	149 (177)	214	20.9^a^	129 (155)	173	11.6^a^	20 (29)	41	41.4^a^
Nov 2020	147 (180)	200	11.1^a^	129 (159)	164	3.1^a^	18 (27)	36	33.3^a^
Dec 2020	150 (182)	164	−9.9	131 (160)	141	−11.9	19 (28)	23	−17.9
Jan 2021	148 (179)	160	−10.6	130 (159)	138	−13.2	17 (26)	22	−15.4
Feb 2021	156 (190)	135	−28.9	135 (165)	122	−26.1	21 (30)	13	−56.7
Mar 2021	154 (188)	189	0.5^a^	131 (162)	155	−4.3	23 (35)	34	−2.9
Apr 2021	164 (198)	134	−32.3	137 (167)	107	−35.9	28 (40)	27	−32.5
May 2021	158 (192)	154	−19.8	134 (166)	128	−22.9	28 (41)	26	−36.6
Relationships									
Jan 2020	57 (70)	67	−4.3	36 (47)	42	−10.6	19 (29)	25	−13.8
Feb 2020	57 (71)	57	−19.7	37 (48)	32	−33.3	20 (29)	25	−13.8
Mar 2020	56 (69)	67	−2.9	36 (46)	42	−8.7	20 (30)	25	−16.7
Apr 2020	56 (70)	39	−44.3	36 (47)	28	−40.4	21 (30)	11	−63.3
May 2020	59 (72)	68	−5.6	37 (48)	43	−10.4	21 (30)	25	−16.7
Jun 2020	59 (73)	36	−50.7	37 (48)	22	−54.2	23 (32)	14	−56.2
Jul 2020	61 (75)	74	−1.3	37 (48)	44	−8.3	24 (34)	30	−11.8
Aug 2020	61 (75)	88	17.3^a^	36 (47)	45	−4.3	25 (35)	43	22.9^a^
Sep 2020	59 (73)	75	2.7^a^	34 (45)	44	−2.2	25 (35)	31	−11.4
Oct 2020	59 (73)	94	28.8^a^	35 (46)	49	6.5^a^	24 (33)	45	36.4^a^
Nov 2020	58 (72)	76	5.6^a^	36 (47)	41	−12.8	21 (30)	35	16.7^a^
Dec 2020	61 (75)	58	−22.7	38 (50)	30	−40.0	22 (31)	28	−9.7
Jan 2021	60 (76)	65	−14.5	37 (48)	40	−16.7	23 (33)	25	−24.2
Feb 2021	60 (76)	56	−26.3	37 (48)	38	−20.8	24 (33)	18	−45.5
Mar 2021	57 (73)	70	−4.1	35 (46)	34	−26.1	24 (34)	36	5.9^a^
Apr 2021	59 (77)	69	−10.4	35 (46)	46	0.0	23 (34)	23	−32.4
May 2021	60 (78)	53	−32.1	36 (48)	34	−29.2	25 (35)	19	−45.7
School									
Jan 2020	28 (39)	38	−2.6	20 (30)	27	−10.0	7 (14)	11	−21.4
Feb 2020	31 (43)	36	−16.3	23 (33)	25	−24.2	8 (15)	11	−26.7
Mar 2020	32 (44)	36	−18.2	24 (34)	28	−17.6	8 (14)	8	−42.9
Apr 2020	32 (45)	27	−40.0	24 (35)	17	−51.4	8 (14)	10	−28.6
May 2020	28 (40)	27	−32.5	21 (32)	21	−34.4	7 (12)	6	−50.0
Jun 2020	26 (37)	29	−21.6	18 (28)	13	−53.6	8 (14)	16	14.3^a^
Jul 2020	24 (35)	24	−31.4	17 (27)	12	−55.6	7 (12)	12	0.0
Aug 2020	29 (41)	54	31.7^a^	21 (32)	28	−12.5	8 (14)	26	85.7^a^
Sep 2020	31 (43)	32	−25.6	23 (33)	18	−45.5	7 (13)	14	7.7^a^
Oct 2020	31 (42)	32	−23.8	23 (33)	21	−36.4	7 (13)	11	−15.4
Nov 2020	29 (41)	43	4.9^a^	20 (31)	29	−6.5	8 (14)	14	0.0
Dec 2020	30 (42)	27	−35.7	21 (31)	14	−54.8	9 (16)	13	−18.8
Jan 2021	34 (46)	27	−41.3	23 (34)	20	−41.2	12 (19)	7	−63.2
Feb 2021	37 (50)	28	−44.0	25 (37)	17	−54.1	12 (20)	11	−45.0
Mar 2021	39 (52)	38	−26.9	28 (39)	25	−35.9	11 (19)	13	−31.6
Apr 2021	36 (49)	28	−42.9	26 (38)	21	−44.7	12 (20)	7	−65.0
May 2021	32 (44)	26	−40.9	22 (33)	17	−48.5	11 (19)	9	−52.6
Others									
Jan 2020	80 (98)	85	−13.3	58 (73)	58	−20.5	22 (31)	27	−12.9
Feb 2020	81 (102)	84	−17.6	58 (74)	65	−12.2	23 (33)	19	−42.4
Mar 2020	83 (104)	99	−4.8	60 (75)	72	−4.0	23 (33)	27	−18.2
Apr 2020	88 (107)	96	−10.3	64 (78)	75	−3.8	25 (34)	21	−38.2
May 2020	84 (104)	86	−17.3	62 (76)	60	−21.1	23 (33)	26	−21.2
Jun 2020	82 (102)	90	−11.8	59 (73)	55	−24.7	23 (33)	35	6.1^a^
Jul 2020	78 (95)	110	15.8^a^	56 (69)	67	−2.9	23 (32)	43	34.4^a^
Aug 2020	80 (99)	124	25.3^a^	57 (70)	87	24.3^a^	23 (33)	37	12.1^a^
Sep 2020	80 (99)	107	8.1^a^	58 (72)	69	−4.2	22 (32)	38	18.8^a^
Oct 2020	81 (97)	121	24.7^a^	60 (73)	66	−9.6	21 (30)	55	83.3^a^
Nov 2020	84 (102)	121	18.6^a^	61 (75)	76	1.3^a^	22 (32)	45	40.6^a^
Dec 2020	85 (103)	98	−4.9	61 (75)	66	−12.0	23 (33)	32	−3.0
Jan 2021	84 (101)	95	−5.9	61 (74)	67	−9.5	24 (33)	28	−15.2
Feb 2021	89 (109)	87	−20.2	63 (77)	61	−20.8	25 (36)	26	−27.8
Mar 2021	92 (112)	97	−13.4	66 (80)	68	−15.0	27 (40)	29	−27.5
Apr 2021	99 (118)	99	−16.1	69 (83)	71	−14.5	31 (44)	28	−36.4
May 2021	97 (121)	105	−13.2	65 (80)	68	−15.0	29 (42)	37	−11.9

^a^ A month with the observed number of suicides exceeding the 95% upper bound of the expected number of suicides for that month. Percentage change was defined as the difference between the observed number of suicides for a month and the 95% upper bound of the expected number of suicides for that month divided by the threshold.

### Reasons for Suicide Among Men

In men, the subcategories that showed 2 months with excess suicide rates were parent-child problems (range, 3.4%-13.0%), physical illness (range, 3.0%-4.8%), physical disability (both months, 5.0%), other debts (range, 1.9%-12.5%), work failure (range, 3.4%-6.9%), work fatigue (range, 2.0%-34.1%), heartbreak (range, 16.7%-17.6%), academic failure (range, 8.3%-16.7%), and loneliness (range, 7.4%-25.0%) (eTable 2 and eFigure 2 in the Supplement). The subcategories that showed 1 month with excess suicide rates were death of a family member (3.8%), unemployment (42.9%), workplace relationships (18.6%), work environment changes (8.3%), infidelity (9.1%), other relationship distress (28.6%), discovery of a crime (4.5%), copycat suicide (14.3%), and other reasons, such as affected by a disaster (27.6%). The highest monthly excess suicide rate was 24.3% for the other category in August 2020 (observed, 87; upper bound of 95% CI for expected number, 70).

### Reasons for Suicide Among Women

The workplace relationships subcategory showed 4 months with excess suicide rates (range, 6.2%-18.2%). The subcategories that showed 3 months with excess suicide rates were poverty (range, 5.9%-26.3%) and work failure (range, 20.0%-40.0%) (eTable 3 and eFigure 3 in the Supplement).

The subcategories that showed 2 months with excess suicide rates were parent-child problems (range, 4.2%-4.5%), marital discord (range, 4.3%-39.1%), other family discords (range, 6.2%-7.1%), child-rearing problems (range, 22.2%-40.0%), physical illness (range, 15.4%-20.4%), depression (range, 15.1%-34.2%), infidelity (range, 7.7%-22.2%), and other relationship distress (range, 13.3%-30.0%). The subcategories showing 1 month with excess suicide rates were caregiving fatigue (25.0%), schizophrenia (26.1%), alcoholism (45.5%), other mental disorders (18.6%), business slump (20.0%), multiple debts (16.7%), work fatigue (13.3%), schoolmate trouble (60.0%), and copycat suicide (12.5%). The highest monthly excess suicide rate was 85.7% for school in August 2020 (observed, 26; upper bound of 95% CI for expected number, 14).

## Discussion

To our knowledge, this was the first study to examine whether the COVID-19 pandemic was associated with changes in reasons for suicide in Japan. Overall, the excess trends for suicides in our study concur with those in prior research.^7,8^ Furthermore, in the early stages of the COVID-19 pandemic, there seems to have been a lack of excessive numbers for suicide worldwide; this may owe to the pulling together phenomenon,^53,54^ wherein a crisis temporally reinforces social bonds. Nonetheless, we observed excess suicide rates around July 2020, during the onset of the second COVID-19 wave in Japan; this wave may have been associated with a diminished sense of social bonding.

The category school demonstrated the highest excess suicide rate in October 2020, although its subcategories did not corroborate this excess. This spike might be because of the accumulated stress from unstable school schedules, school closures, and the sudden shift to online education, all of which started in March 2020.^55^ Considering that the younger generation experiences high levels of stress and is at risk for suicide,^35,56^ there is a critical need for educational interventions within schools on the mental health effects of the COVID-19 pandemic, which have shown to be effective.^57^

We observed that women showed excess suicide rates across all categories. Men did not have an excess rate for school, although they did for all other categories. Despite these gender differences, there were excess suicide rates in almost all categories, indicating that the COVID-19 pandemic evoked multilayered psychological distresses in Japan; this concurs with prior international research.^20,21,22,23^

### Reasons for Suicide in Men

Our results suggested that, generally, work-related stress (eg, work failure, fatigue) was associated with suicide in men. This is consistent with previous studies,^20,40,58^ although these studies did not inspect gender differences. In Japan in 2016, men owned 80% of the households;^60^ in 2015, male full-time employees tended to have a median monthly income 25.7% higher than that of their female counterparts.^59^ Thus, men remain the main breadwinners of their households nationwide. The economic impact of the COVID-19 pandemic seems to have been severe enough for many men to resort to suicide. Thus, relevant stakeholders (eg, employment assistance programs, company-based employee benefit programs) and occupational health services need to provide men in vulnerable work-related circumstances with social and mental health support via telecommunication or online services.^60,61^

We observed higher suicide rates with an unknown reason among men, which may be explained by gender differences in help-seeking behavior. Globally, men tend to engage in less help-seeking behaviors for psychological hardships than women.^15,16,17^ In Japan, while men tended to not leave suicide notes, women did leave them.^62^ In our results for men, the subcategories of heartbreak, relationship distress, and loneliness showed rates that were significantly higher than the estimations for some months. In a systematic review,^21^ psychosocial factors (eg, social isolation) were shown to be significant risk factors of suicide, a finding that concurs with our evidence. Therefore, we see the need for suicide prevention campaigns tailored to men, such as those using male role models,^63^ that promote mental health support.

### Reasons for Suicide in Women

Previous studies reporting higher suicide rates in women than men speculated reasons for suicide that included job loss and caregiver roles.^11,12^ In our study, we observed that both these factors and health problems may be associated with substantial burden in Japanese women. Furthermore, women showed excess suicide rates across several consecutive months, demonstrating that the COVID-19 pandemic may have burdened several aspects of their lives. Our findings for women correspond to those in previous studies,^18,19,20,21,38^ although we provide more detailed data on the potential reasons for their suicide. It is possible that school closures, telecommuting, an increase in caregiver role, and restrictions in access to health services were associated with women spending more time with family members; this may have been associated with the excess suicide rates we observed by exacerbating parent-child problems, other family discords, child-rearing problems, and caregiving fatigue.

We also observed excess suicide rates associated with depression, schizophrenia, alcoholism, and other mental disorders in women. Research shows that preexisting mental health problems are a risk factor for suicide.^17^ As suicide tends to be associated with various factors,^17^ we suggest that health care professionals inquire about women’s life changes since the onset of the COVID-19 pandemic, provide psychosocial support, assess the risk of suicide, and consider referral to a psychiatrist when relevant.

### Copycat Suicide

From 1989 to 2010, the 10 days following a media report of the suicide of a well-known Japanese figure tended to accompany an increase in the number of copycat suicides.^64^ Considering this and the excess suicide rates we observed for copycat suicide in women, our results seem to indicate the possibility of the Werther effect in Japanese women. Nonetheless, our results for copycat suicides in men showed excess suicide rates throughout April, a period inconsistent with that for women. A systematic review on this topic^65^ showed that age and gender have a strong modeling effect on suicide. We could not identify possible reasons for this gender difference.

Regarding the prevention of copycat suicides, research shows that this can be operationalized through media cooperation^43^; 2 studies^66,67^ demonstrated that people searched for suicide-related information on the internet (ie, through search engines and social media) during the early stages of the COVID-19 pandemic. Online news and social media platforms should be cautious when reporting suicide-related information.

### Limitations

This study has limitations. Our data set has potential bias and errors owing to the fact that those who die by suicide cannot report on the actual reasoning behind their act. However, we did use data from national agency sources, which we deemed the most reliable sources at the time of this research.

Second, we excluded 30% of our data on suicide deaths, as these were categorized under the reason unknown. Moreover, our χ^2^ analysis comparing reason-identified suicides and unknown reason suicides by gender yielded significant results. Thus, if the reasons in the missing data greatly differ from those in the reason-identified suicides we used, the exclusion of this portion of the data may have affected our findings.

Third, there is a potential lack of accuracy for our data on reasons for suicide; nonetheless, there are no current scientific measures that can accurately determine the true reasons behind a suicide, so we still deem the governmental data set we used the most reliable source among those currently available.

Fourth, although the Farrington algorithm is a well-established methodology, it has yet to receive an extension that enables including covariates; this hindered our ability to include geographical factors in the model. Furthermore, it is possible that factors other than the COVID-19 pandemic were associated with the suicide cases we analyzed.

Fifth, as suicide is also influenced by culture and religion,^17^ there are clear limitations regarding the generalizability of our results. Despite these limits, we believe that our findings shed light on the reasons for suicide amid the COVID-19 pandemic.

## Conclusions

This study found that the COVID-19 pandemic may be associated with various changes in the reasons for suicide in Japan. We observed excess suicide rates in all categories, albeit with differences in subcategories by gender. In women, the categories of family, health, work, and other showed excess suicide rates that lasted from 5 to 6 consecutive months. We hope that our data are used as a basis for the development of suicide prevention interventions and programs.

## Notes

Supplement. **eFigure 1.** Number of Suicides by the 7 Categories of Reasons for Suicide **eFigure 2.** Subcategories of Reasons for Suicide With Rates Exceeding the Upper Bound of the 95% CI for a Month Among Men **eFigure 3.** Subcategories of Reasons for Suicide With Rates Exceeding the Upper Bound of the 95% CI for a Month Among Women **eTable 1.** Number of Suicides by Age and Subcategory, January 2020 to May 2021 **eTable 2. **Expected and Observed Number of Monthly Suicides and Percentage Change From January 2020 to May 2021 by 52 Subcategories Among Men **eTable 3.** Expected and Observed Number of Monthly Suicides and Percentage Change From January 2020 to May 2021 by 52 Subcategories Among Women
