# Error in Results

**DOI:** 10.1001/jamanetworkopen.2022.3161

**Published:** 2022-02-22

In the Original Investigation titled “Reasons for Suicide During the COVID-19 Pandemic in Japan,”^1^ published January 31, 2022, there was an error in the Results section. In the section describing subcategories of reason for suicide that had 1 month with an excess suicide rate among women, the excess suicide rate for work fatigue should have read 133.3%. This article has been corrected.^1^
