# Supplementary material for: Reasons for Suicide During the COVID-19 Pandemic in Japan
Source: JAMA Netw Open. 2022 Jan 31;5(1):e2145870. doi: 10.1001/jamanetworkopen.2021.45870 (PMC8804915; doi:10.1001/jamanetworkopen.2021.45870)
Supplement: Supplement. — eFigure 1. Number of Suicides by the 7 Categories of Reasons for Suicide eFigure 2. Subcategories of Reasons for Suicide With Rates Exceeding the Upper Bound of the 95% CI for a Month Among Men eFigure 3. Subcategories of Reasons for Suicide With Rates Exceeding the Upper Bound of the 95% CI for a Month Among Women eTable 1. Number of Suicides by Age and Subcategory, January 2020 to May 2021 eTable 2. Expected and Observed Number of Monthly Suicides and Percentage Change From January 2020 to May 2021 by 52 Subcategories Among Men eTable 3. Expected and Observed Number of Monthly Suicides and Percentage Change From January 2020 to May 2021 by 52 Subcategories Among Women [file jamanetwopen-e2145870-s001.pdf]

Supplemental Online Content

Koda M, Harada N, Eguchi A, Nomura S, Ishida Y. Reasons for suicide during the COVID-19 pandemic in Japan. *JAMA Netw Open.* 2022;5(1):e2145870. doi:10.1001/jamanetworkopen.2021.45870

eFigure 1. Number of Suicides by the 7 Categories of Reasons for Suicide

eFigure 2. Subcategories of Reasons for Suicide With Rates Exceeding the Upper Bound of the 95% CI for a Month Among Men

eFigure 3. Subcategories of Reasons for Suicide With Rates Exceeding the Upper Bound of the 95% CI for a Month Among Women

eTable 1. Number of Suicides by Age and Subcategory, January 2020 to May 2021

eTable 2. Expected and Observed Number of Monthly Suicides and Percentage Change From January 2020 to May 2021 by 52 Subcategories Among Men

eTable 3. Expected and Observed Number of Monthly Suicides and Percentage Change From January 2020 to May 2021 by 52 Subcategories Among Women

This supplemental material has been provided by the authors to give readers additional information about their work.

© 2022 Koda M et al. *JAMA Network Open.*

eFigure 1. Number of Suicides by the 7 Categories of Reasons for Suicide

We used the Farrington algorithm in a Quasi-Poisson regression model for the seven categories. Specifically, we set the expected number of suicides for a month and the upper bound of the 95% confidence interval for the expected number of suicides for that month based on data from the past five years for that given month and the months immediately before and after (i.e., data from March 2020 versus data among February, March, and April between 2015 and 2019) . The vertical axis shows the number of suicides, and the horizontal axis shows the time series. The upper bound is shown in yellow, and months with a high number of suicides are shown in pink.

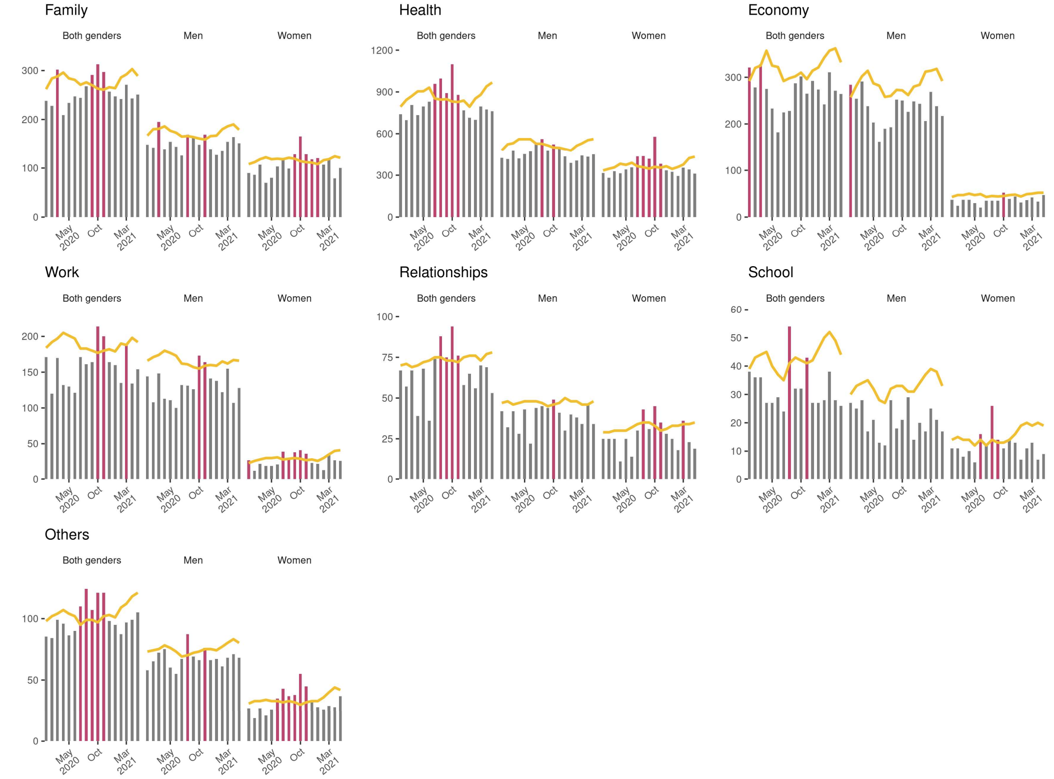

© 2022 Koda M et al. *JAMA Network Open.*

eFigure 2. Subcategories of Reasons for Suicide With Rates Exceeding the Upper Bound of the 95% CI for a Month Among Men

We used the Farrington algorithm in a Quasi-Poisson regression model for each subcategory among men. Specifically, we set the expected number of suicides for a month and the upper bound of the 95% confidence interval for the expected number of suicides for that month based on data from the past year (i.e., data from January 2020 and May 2021 versus between January 2019 and June 2020). The vertical axis shows the number of suicides, and the horizontal axis shows the time series. The upper bound is shown in yellow, and months with a high number of suicides are shown in pink.

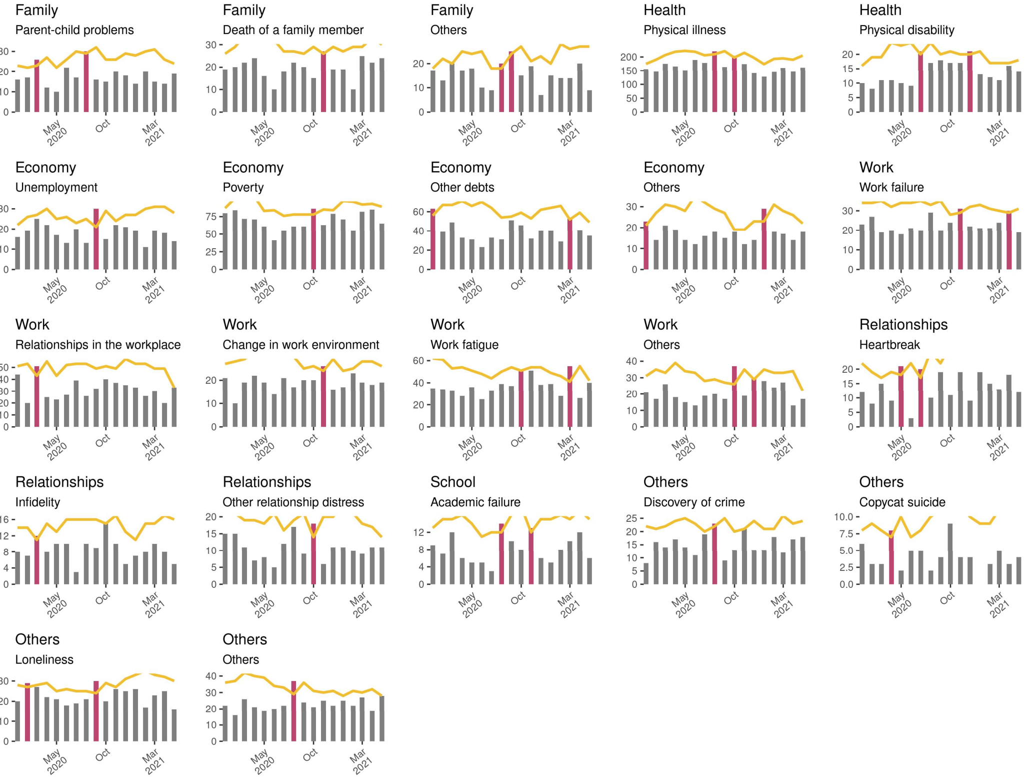

© 2022 Koda M et al. *JAMA Network Open.*

eFigure 3. Subcategories of Reasons for Suicide With Rates Exceeding the Upper Bound of the 95% CI for a Month Among Women

We used the Farrington algorithm in a Quasi-Poisson regression model for each subcategory among women. Specifically, we set the expected number of suicides for a month and the upper bound of the 95% confidence interval for the expected number of suicides for that month based on data from the past year (i.e., data from January 2020 and May 2021 versus from January 2019 and June 2020). The upper bound is shown in yellow, and months with a high number of suicides are shown in pink.

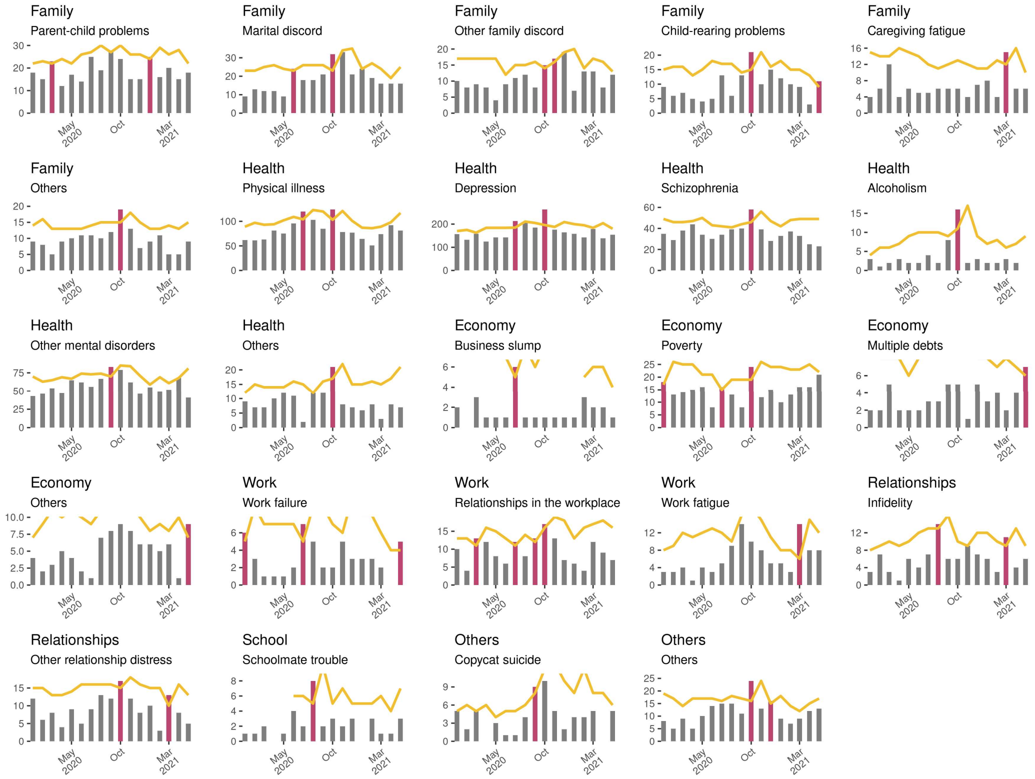

eTable 1. Number of Suicides by Age and Subcategory, January 2020 to May 2021

	Age group (No)									
	Total	-19	20-29	30-39	40-49	50-59	60-69	70-79	80-	Unknown
**Family – Parent–child problems**										
**Both**	619	86	111	66	84	88	48	73	63	0
**Men**	301	41	56	37	40	39	29	32	27	0
**Women**	318	45	55	29	44	49	19	41	36	0
**Family - Marital discord**										
**Both**	1,092	1	79	235	293	206	127	109	42	0
**Men**	779	1	55	177	207	147	86	78	28	0
**Women**	313	0	24	58	86	59	41	31	14	0
**Family - Other family discords**										
**Both**	443	21	49	73	88	74	51	47	40	0
**Men**	260	9	28	52	57	43	28	24	19	0
**Women**	183	12	21	21	31	31	23	23	21	0
**Family - Death of a family member**										
**Both**	587	6	24	40	73	113	96	124	111	0
**Men**	332	3	16	26	46	63	51	62	65	0
**Women**	255	3	8	14	27	50	45	62	46	0

© 2022 Koda M et al. *JAMA Network Open.*

	Age group (No)									
	Total	-19	20-29	30-39	40-49	50-59	60-69	70-79	80-	Unknown
**Family - Pessimism about the future of the family**										
**Both**	583	15	36	49	98	92	102	110	81	0
**Men**	331	10	19	25	64	42	62	59	50	0
**Women**	252	5	17	24	34	50	40	51	31	0
**Family - Abuse from family**										
**Both**	184	45	40	15	24	13	6	21	20	0
**Men**	124	27	29	12	17	6	5	14	14	0
**Women**	60	18	11	3	7	7	1	7	6	0
**Family - Child-rearing problems**										
**Both**	189	0	29	69	61	21	9	0	0	0
**Men**	30	0	5	4	13	5	3	0	0	0
**Women**	159	0	24	65	48	16	6	0	0	0
**Family - Abuse**										
**Both**	3	1	1	0	0	0	0	1	0	0
**Men**	1	1	0	0	0	0	0	0	0	0
**Women**	2	0	1	0	0	0	0	1	0	0
**Family - Caregiving fatigue**										
**Both**	257	0	3	4	17	70	66	56	41	0

© 2022 Koda M et al. *JAMA Network Open.*

	Age group (No)									
	Total	-19	20-29	30-39	40-49	50-59	60-69	70-79	80-	Unknown
**Men**	147	0	3	4	8	39	40	26	27	0
**Women**	110	0	0	0	9	31	26	30	14	0
**Family - Others**										
**Both**	425	16	43	43	65	73	42	65	78	0
**Men**	262	12	35	27	45	35	20	42	46	0
**Women**	163	4	8	16	20	38	22	23	32	0
**Health - Physical illness**										
**Both**	4,192	16	79	121	290	452	768	1,279	1,186	1
**Men**	2,805	9	45	80	171	281	528	913	777	1
**Women**	1,387	7	34	41	119	171	240	366	409	0
**Health - Depression**										
**Both**	5,570	87	533	676	1,043	1,089	914	848	379	1
**Men**	2,728	34	236	350	561	585	453	344	164	1
**Women**	2,842	53	297	326	482	504	461	504	215	0
**Health - Schizophrenia**										
**Both**	1,194	21	143	235	314	231	138	85	26	1
**Men**	595	8	69	118	165	118	68	38	10	1
**Women**	599	13	74	117	149	113	70	47	16	0

© 2022 Koda M et al. *JAMA Network Open.*

	Age group (No)									
	Total	-19	20-29	30-39	40-49	50-59	60-69	70-79	80-	Unknown
**Health - Alcoholism**										
**Both**	215	1	8	26	54	66	36	16	8	0
**Men**	158	0	4	14	36	52	32	15	5	0
**Women**	57	1	4	12	18	14	4	1	3	0
**Health - Drug and substance abuse**										
**Both**	48	1	6	11	16	6	6	2	0	0
**Men**	28	1	1	4	14	3	5	0	0	0
**Women**	20	0	5	7	2	3	1	2	0	0
**Health - Other mental disorders**										
**Both**	2,002	90	301	266	347	309	207	278	202	2
**Men**	1,027	31	154	160	178	170	106	125	101	2
**Women**	975	59	147	106	169	139	101	153	101	0
**Health - Physical disability**										
**Both**	350	4	8	11	24	42	53	101	106	1
**Men**	236	3	7	9	17	31	35	64	70	0
**Women**	114	1	1	2	7	11	18	37	36	1
**Health - Others**										
**Both**	369	17	32	30	42	44	50	64	90	0

© 2022 Koda M et al. *JAMA Network Open.*

	Age group (No)									
	Total	-19	20-29	30-39	40-49	50-59	60-69	70-79	80-	Unknown
**Men**	219	12	23	20	25	23	29	35	52	0
**Women**	150	5	9	10	17	21	21	29	38	0
**Economy - Bankruptcy**										
**Both**	25	0	1	2	8	6	5	3	0	0
**Men**	21	0	1	2	7	4	4	3	0	0
**Women**	4	0	0	0	1	2	1	0	0	0
**Economy - Business slump**										
**Both**	389	0	12	42	96	103	95	37	3	1
**Men**	361	0	12	38	87	95	90	35	3	1
**Women**	28	0	0	4	9	8	5	2	0	0
**Economy - Unemployment**										
**Both**	358	1	30	51	99	119	40	18	0	0
**Men**	314	1	25	42	92	103	39	12	0	0
**Women**	44	0	5	9	7	16	1	6	0	0
**Economy - Job failure**										
**Both**	263	3	111	55	38	45	8	3	0	0
**Men**	215	2	89	45	33	37	7	2	0	0
**Women**	48	1	22	10	5	8	1	1	0	0

© 2022 Koda M et al. *JAMA Network Open.*

	Age group (No)									
	Total	-19	20-29	30-39	40-49	50-59	60-69	70-79	80-	Unknown
**Economy - Poverty**										
**Both**	1,420	6	101	173	281	373	303	143	37	3
**Men**	1,172	3	84	140	235	312	265	108	23	2
**Women**	248	3	17	33	46	61	38	35	14	1
**Economy - Multiple debts**										
**Both**	863	1	153	177	205	175	114	33	5	0
**Men**	806	1	144	165	192	164	109	28	3	0
**Women**	57	0	9	12	13	11	5	5	2	0
**Economy - Joint guarantee**										
**Both**	19	0	0	4	3	3	5	3	1	0
**Men**	16	0	0	3	2	3	5	3	0	0
**Women**	3	0	0	1	1	0	0	0	1	0
**Economy - Other debts**										
**Both**	748	4	109	133	166	163	119	50	4	0
**Men**	669	4	93	125	147	146	109	43	2	0
**Women**	79	0	16	8	19	17	10	7	2	0
**Economy - Debt collection trouble**										
**Both**	74	1	7	18	14	20	13	0	1	0

© 2022 Koda M et al. *JAMA Network Open.*

	Age group (No)									
	Total	-19	20-29	30-39	40-49	50-59	60-69	70-79	80-	Unknown
**Men**	63	1	4	16	11	19	12	0	0	0
**Women**	11	0	3	2	3	1	1	0	1	0
**Economy - Suicide for insurance**										
**Both**	41	0	3	2	7	17	8	4	0	0
**Men**	35	0	3	1	5	15	7	4	0	0
**Women**	6	0	0	1	2	2	1	0	0	0
**Economy - Others**										
**Both**	378	4	55	56	60	81	55	42	25	0
**Men**	292	2	44	45	50	62	38	34	17	0
**Women**	86	2	11	11	10	19	17	8	8	0
**Work - Work failure**										
**Both**	440	11	99	73	96	103	44	12	2	0
**Men**	389	9	78	69	85	95	41	10	2	0
**Women**	51	2	21	4	11	8	3	2	0	0
**Work – Workplace relationships**										
**Both**	702	14	161	177	179	138	29	3	1	0
**Men**	541	11	106	133	147	113	27	3	1	0
**Women**	161	3	55	44	32	25	2	0	0	0

	Age group (No)									
	Total	-19	20-29	30-39	40-49	50-59	60-69	70-79	80-	Unknown
**Work - Work environment changes**										
**Both**	386	6	72	76	103	94	28	6	1	0
**Men**	321	5	53	65	84	82	25	6	1	0
**Women**	65	1	19	11	19	12	3	0	0	0
**Work - Work fatigue**										
**Both**	736	10	147	147	200	160	62	9	1	0
**Men**	629	9	119	118	175	143	55	9	1	0
**Women**	107	1	28	29	25	17	7	0	0	0
**Work - Others**										
**Both**	426	6	97	68	111	81	39	19	5	0
**Men**	361	4	74	60	94	76	32	17	4	0
**Women**	65	2	23	8	17	5	7	2	1	0
**Relationships - Marriage**										
**Both**	70	0	23	21	16	9	1	0	0	0
**Men**	41	0	10	13	12	5	1	0	0	0
**Women**	29	0	13	8	4	4	0	0	0	0
**Relationships - Heartbreak**										
**Both**	355	42	137	97	44	24	10	1	0	0

	Age group (No)									
	Total	-19	20-29	30-39	40-49	50-59	60-69	70-79	80-	Unknown
**Men**	233	28	81	71	27	18	8	0	0	0
**Women**	122	14	56	26	17	6	2	1	0	0
**Relationships - Infidelity**										
**Both**	248	1	38	86	64	40	12	6	1	0
**Men**	145	0	12	55	37	24	12	4	1	0
**Women**	103	1	26	31	27	16	0	2	0	0
**Relationships - Other relationship distress**										
**Both**	340	29	122	84	76	21	4	3	1	0
**Men**	186	15	58	49	44	13	3	3	1	0
**Women**	154	14	64	35	32	8	1	0	0	0
**Relationships - Others**										
**Both**	99	11	23	32	14	9	1	8	1	0
**Men**	49	4	10	17	4	7	1	6	0	0
**Women**	50	7	13	15	10	2	0	2	1	0
**School - Admissions**										
**Both**	48	34	11	3	0	0	0	0	0	0
**Men**	33	22	8	3	0	0	0	0	0	0
**Women**	15	12	3	0	0	0	0	0	0	0

© 2022 Koda M et al. *JAMA Network Open.*

	Age group (No)									
	Total	-19	20-29	30-39	40-49	50-59	60-69	70-79	80-	Unknown
**School - Academic path**										
**Both**	181	83	95	3	0	0	0	0	0	0
**Men**	118	48	67	3	0	0	0	0	0	0
**Women**	63	35	28	0	0	0	0	0	0	0
**School - Academic failure**										
**Both**	183	87	92	4	0	0	0	0	0	0
**Men**	139	61	75	3	0	0	0	0	0	0
**Women**	44	26	17	1	0	0	0	0	0	0
**School – Issues with teachers**										
**Both**	8	6	2	0	0	0	0	0	0	0
**Men**	5	3	2	0	0	0	0	0	0	0
**Women**	3	3	0	0	0	0	0	0	0	0
**School - Bullying**										
**Both**	11	11	0	0	0	0	0	0	0	0
**Men**	3	3	0	0	0	0	0	0	0	0
**Women**	8	8	0	0	0	0	0	0	0	0
**School - Schoolmate trouble**										
**Both**	58	38	18	2	0	0	0	0	0	0

© 2022 Koda M et al. *JAMA Network Open.*

	Age group (No)									
	Total	-19	20-29	30-39	40-49	50-59	60-69	70-79	80-	Unknown
**Men**	21	10	10	1	0	0	0	0	0	0
**Women**	37	28	8	1	0	0	0	0	0	0
**School - Others**										
**Both**	63	44	17	1	1	0	0	0	0	0
**Men**	34	24	9	1	0	0	0	0	0	0
**Women**	29	20	8	0	1	0	0	0	0	0
**Others - Discovery of a crime**										
**Both**	291	9	27	57	85	58	26	21	8	0
**Men**	256	8	24	50	75	52	23	18	6	0
**Women**	35	1	3	7	10	6	3	3	2	0
**Others - Victim of a crime**										
**Both**	14	1	2	4	2	1	2	1	1	0
**Men**	5	0	0	0	2	1	1	1	0	0
**Women**	9	1	2	4	0	0	1	0	1	0
**Others - Copycat suicide**										
**Both**	135	0	13	11	24	28	18	22	19	0
**Men**	70	0	4	5	12	17	7	13	12	0
**Women**	65	0	9	6	12	11	11	9	7	0

© 2022 Koda M et al. *JAMA Network Open.*

	Age group (No)									
	Total	-19	20-29	30-39	40-49	50-59	60-69	70-79	80-	Unknown
**Others - Loneliness**										
**Both**	608	22	65	44	74	84	86	101	132	0
**Men**	385	10	43	30	57	61	56	62	66	0
**Women**	223	12	22	14	17	23	30	39	66	0
**Others - Neighborhood trouble**										
**Both**	65	0	1	4	8	6	16	19	11	0
**Men**	39	0	0	0	4	5	13	14	3	0
**Women**	26	0	1	4	4	1	3	5	8	0
**Others - Others**										
**Both**	591	50	140	85	75	52	57	70	62	0
**Men**	396	29	97	63	45	37	38	50	37	0
**Women**	195	21	43	22	30	15	19	20	25	0

© 2022 Koda M et al. *JAMA Network Open.*

eTable 2. **Expected and Observed Number of Monthly Suicides and Percentage Change From January 2020 to May 2021 by 52 Subcategories Among Men**

	Expected number of monthly suicides (No)	95% upper bound of the expected number of monthly suicides (No)	Observed number of suicides (No)	Percent change (%)
**Family – Parent–child problems**				
**2020-01**	16	23	16	-30.4
**2020-02**	15	22	17	-22.7
**2020-03**	16	23	26	13.0 *
**2020-04**	18	27	12	-55.6
**2020-05**	15	22	10	-54.5
**2020-06**	16	26	22	-15.4
**2020-07**	19	30	17	-43.3
**2020-08**	19	29	30	3.4 *
**2020-09**	20	32	16	-50.0
**2020-10**	16	26	15	-42.3
**2020-11**	16	26	20	-23.1
**2020-12**	17	29	18	-37.9
**2021-01**	18	28	14	-50.0
**2021-02**	18	30	20	-33.3
**2021-03**	19	31	15	-51.6
**2021-04**	16	26	14	-46.2
**2021-05**	15	24	19	-20.8
**Family - Marital discord**				
**2020-01**	52	66	47	-28.8
**2020-02**	50	63	47	-25.4
**2020-03**	49	62	58	-6.5
**2020-04**	50	63	47	-25.4
**2020-05**	48	61	50	-18.0
**2020-06**	47	60	56	-6.7

© 2022 Koda M et al. *JAMA Network Open.*

	Expected number of monthly suicides (No)	95% upper bound of the expected number of monthly suicides (No)	Observed number of suicides (No)	Percent change (%)
**2020-07**	50	62	36	-41.9
**2020-08**	43	57	44	-22.8
**2020-09**	44	57	49	-14.0
**2020-10**	44	58	50	-13.8
**2020-11**	45	58	44	-24.1
**2020-12**	47	60	48	-20.0
**2021-01**	49	62	34	-45.2
**2021-02**	47	59	42	-28.8
**2021-03**	48	62	43	-30.6
**2021-04**	49	63	44	-30.2
**2021-05**	49	62	40	-35.5
**Family - Other family discords**				
**2020-01**	18	28	18	-35.7
**2020-02**	18	30	16	-46.7
**2020-03**	14	23	17	-26.1
**2020-04**	15	22	7	-68.2
**2020-05**	14	26	14	-46.2
**2020-06**	18	30	11	-63.3
**2020-07**	17	27	13	-51.9
**2020-08**	14	23	10	-56.5
**2020-09**	12	21	17	-19.0
**2020-10**	12	19	16	-15.8
**2020-11**	14	22	20	-9.1
**2020-12**	16	27	19	-29.6
**2021-01**	18	26	16	-38.5
**2021-02**	17	25	12	-52.0
**2021-03**	13	20	18	-10.0
**2021-04**	14	21	19	-9.5

© 2022 Koda M et al. *JAMA Network Open.*

	Expected number of monthly suicides (No)	95% upper bound of the expected number of monthly suicides (No)	Observed number of suicides (No)	Percent change (%)
**2021-05**	13	20	17	-15.0
**Family - Death of a family member**				
**2020-01**	18	26	19	-26.9
**2020-02**	20	29	20	-31.0
**2020-03**	24	34	22	-35.3
**2020-04**	26	36	24	-33.3
**2020-05**	27	37	16	-56.8
**2020-06**	23	32	10	-68.8
**2020-07**	17	27	18	-33.3
**2020-08**	18	27	22	-18.5
**2020-09**	18	26	20	-23.1
**2020-10**	20	29	15	-48.3
**2020-11**	18	26	27	3.8 *
**2020-12**	21	29	19	-34.5
**2021-01**	18	27	19	-29.6
**2021-02**	20	29	10	-65.5
**2021-03**	19	29	25	-13.8
**2021-04**	22	33	22	-33.3
**2021-05**	18	30	24	-20.0
**Family - Pessimism about the future of the family**				
**2020-01**	19	31	20	-35.5
**2020-02**	24	34	12	-64.7
**2020-03**	19	31	31	0.0
**2020-04**	25	37	21	-43.2
**2020-05**	20	32	21	-34.4
**2020-06**	21	33	21	-36.4
**2020-07**	19	28	20	-28.6
**2020-08**	21	31	25	-19.4

© 2022 Koda M et al. *JAMA Network Open.*

	Expected number of monthly suicides (No)	95% upper bound of the expected number of monthly suicides (No)	Observed number of suicides (No)	Percent change (%)
**2020-09**	21	32	17	-46.9
**2020-10**	19	28	14	-50.0
**2020-11**	16	26	21	-19.2
**2020-12**	20	30	10	-66.7
**2021-01**	14	21	15	-28.6
**2021-02**	20	31	19	-38.7
**2021-03**	21	33	20	-39.4
**2021-04**	23	33	22	-33.3
**2021-05**	21	30	22	-26.7
**Family - Abuse from family**				
**2020-01**	7	13	4	-69.2
**2020-02**	8	14	10	-28.6
**2020-03**	8	14	11	-21.4
**2020-04**	9	15	6	-60.0
**2020-05**	7	13	9	-30.8
**2020-06**	9	15	7	-53.3
**2020-07**	8	14	6	-57.1
**2020-08**	7	12	11	-8.3
**2020-09**	8	14	7	-50.0
**2020-10**	7	13	11	-15.4
**2020-11**	8	14	5	-64.3
**2020-12**	5	9	7	-22.2
**2021-01**	7	12	4	-66.7
**2021-02**	7	14	6	-57.1
**2021-03**	8	14	4	-71.4
**2021-04**	8	13	8	-38.5
**2021-05**	8	13	8	-38.5
**Family - Child-rearing problems**				

© 2022 Koda M et al. *JAMA Network Open.*

	Expected number of monthly suicides (No)	95% upper bound of the expected number of monthly suicides (No)	Observed number of suicides (No)	Percent change (%)
**2020-01**	5	9	1	-88.9
**2020-02**	3	8	0	-100.0
**2020-03**	2	6	0	-100.0
**2020-04**	-	-	0	-
**2020-05**	-	-	0	-
**2020-06**	-	-	1	-
**2020-07**	-	-	1	-
**2020-08**	-	-	1	-
**2020-09**	1	5	3	-40.0
**2020-10**	2	6	4	-33.3
**2020-11**	3	9	3	-66.7
**2020-12**	2	7	2	-71.4
**2021-01**	2	6	3	-50.0
**2021-02**	1	6	1	-83.3
**2021-03**	0	5	2	-60.0
**2021-04**	1	5	3	-40.0
**2021-05**	1	5	5	0.0
**Family - Abuse**				
**2020-01**	-	-	0	-
**2020-02**	-	-	0	-
**2020-03**	-	-	0	-
**2020-04**	-	-	0	-
**2020-05**	-	-	0	-
**2020-06**	-	-	0	-
**2020-07**	-	-	0	-
**2020-08**	-	-	0	-
**2020-09**	-	-	0	-
**2020-10**	-	-	0	-

© 2022 Koda M et al. *JAMA Network Open.*

	Expected number of monthly suicides (No)	95% upper bound of the expected number of monthly suicides (No)	Observed number of suicides (No)	Percent change (%)
**2020-11**	-	-	0	-
**2020-12**	-	-	0	-
**2021-01**	-	-	0	-
**2021-02**	-	-	0	-
**2021-03**	-	-	0	-
**2021-04**	-	-	1	-
**2021-05**	-	-	0	-
**Family - Caregiving fatigue**				
**2020-01**	10	17	6	-64.7
**2020-02**	13	22	7	-68.2
**2020-03**	13	20	10	-50.0
**2020-04**	14	21	5	-76.2
**2020-05**	11	19	16	-15.8
**2020-06**	14	21	6	-71.4
**2020-07**	12	21	6	-71.4
**2020-08**	11	23	6	-73.9
**2020-09**	11	20	9	-55.0
**2020-10**	10	17	8	-52.9
**2020-11**	9	18	10	-44.4
**2020-12**	7	14	9	-35.7
**2021-01**	7	12	7	-41.7
**2021-02**	8	14	11	-21.4
**2021-03**	8	15	13	-13.3
**2021-04**	11	18	11	-38.9
**2021-05**	10	17	7	-58.8
**Family - Others**				
**2020-01**	11	18	17	-5.6
**2020-02**	14	22	13	-40.9

© 2022 Koda M et al. *JAMA Network Open.*

	Expected number of monthly suicides (No)	95% upper bound of the expected number of monthly suicides (No)	Observed number of suicides (No)	Percent change (%)
**2020-03**	13	20	20	0.0
**2020-04**	17	25	17	-32.0
**2020-05**	16	24	18	-25.0
**2020-06**	17	25	10	-60.0
**2020-07**	12	18	9	-50.0
**2020-08**	12	18	20	11.1 *
**2020-09**	14	24	25	4.2 *
**2020-10**	16	27	15	-44.4
**2020-11**	12	21	19	-9.5
**2020-12**	14	23	7	-69.6
**2021-01**	11	20	15	-25.0
**2021-02**	16	28	14	-50.0
**2021-03**	16	26	14	-46.2
**2021-04**	17	27	20	-25.9
**2021-05**	16	27	9	-66.7
**Health - Physical illness**				
**2020-01**	153	175	156	-10.9
**2020-02**	162	190	146	-23.2
**2020-03**	172	207	175	-15.5
**2020-04**	195	219	166	-24.2
**2020-05**	189	222	152	-31.5
**2020-06**	185	219	189	-13.7
**2020-07**	183	206	178	-13.6
**2020-08**	184	210	220	4.8 *
**2020-09**	188	221	164	-25.8
**2020-10**	175	198	204	3.0 *
**2020-11**	177	215	174	-19.1
**2020-12**	165	197	141	-28.4

© 2022 Koda M et al. *JAMA Network Open.*

	Expected number of monthly suicides (No)	95% upper bound of the expected number of monthly suicides (No)	Observed number of suicides (No)	Percent change (%)
**2021-01**	149	174	128	-26.4
**2021-02**	151	193	145	-24.9
**2021-03**	158	195	160	-17.9
**2021-04**	163	190	146	-23.2
**2021-05**	163	205	161	-21.5
**Health - Depression**				
**2020-01**	157	185	151	-18.4
**2020-02**	164	198	136	-31.3
**2020-03**	175	213	173	-18.8
**2020-04**	187	216	139	-35.6
**2020-05**	172	215	159	-26.0
**2020-06**	175	207	162	-21.7
**2020-07**	179	209	186	-11.0
**2020-08**	184	216	185	-14.4
**2020-09**	178	206	156	-24.3
**2020-10**	161	184	180	-2.2
**2020-11**	165	195	179	-8.2
**2020-12**	158	186	170	-8.6
**2021-01**	152	176	136	-22.7
**2021-02**	149	173	137	-20.8
**2021-03**	146	177	160	-9.6
**2021-04**	158	184	155	-15.8
**2021-05**	154	175	164	-6.3
**Health - Schizophrenia**				
**2020-01**	30	41	33	-19.5
**2020-02**	34	44	36	-18.2
**2020-03**	36	47	32	-31.9
**2020-04**	40	51	27	-47.1

© 2022 Koda M et al. *JAMA Network Open.*

	Expected number of monthly suicides (No)	95% upper bound of the expected number of monthly suicides (No)	Observed number of suicides (No)	Percent change (%)
**2020-05**	38	51	40	-21.6
**2020-06**	43	55	33	-40.0
**2020-07**	39	50	39	-22.0
**2020-08**	40	51	37	-27.5
**2020-09**	34	45	45	0.0
**2020-10**	37	48	29	-39.6
**2020-11**	31	41	39	-4.9
**2020-12**	34	45	30	-33.3
**2021-01**	32	42	35	-16.7
**2021-02**	34	45	34	-24.4
**2021-03**	32	43	35	-18.6
**2021-04**	33	44	31	-29.5
**2021-05**	33	43	40	-7.0
**Health - Alcoholism**				
**2020-01**	9	15	8	-46.7
**2020-02**	10	17	17	0.0
**2020-03**	12	18	10	-44.4
**2020-04**	10	18	8	-55.6
**2020-05**	10	19	6	-68.4
**2020-06**	9	15	7	-53.3
**2020-07**	9	16	8	-50.0
**2020-08**	9	18	13	-27.8
**2020-09**	13	20	11	-45.0
**2020-10**	12	21	6	-71.4
**2020-11**	8	16	13	-18.8
**2020-12**	8	15	10	-33.3
**2021-01**	10	18	10	-44.4
**2021-02**	11	18	8	-55.6

© 2022 Koda M et al. *JAMA Network Open.*

	Expected number of monthly suicides (No)	95% upper bound of the expected number of monthly suicides (No)	Observed number of suicides (No)	Percent change (%)
**2021-03**	11	17	8	-52.9
**2021-04**	8	14	10	-28.6
**2021-05**	8	13	5	-61.5
**Health - Drug and substance abuse**				
**2020-01**	-	-	3	-
**2020-02**	-	-	1	-
**2020-03**	2	7	3	-57.1
**2020-04**	2	7	0	-100.0
**2020-05**	1	5	1	-80.0
**2020-06**	1	5	2	-60.0
**2020-07**	1	5	2	-60.0
**2020-08**	1	6	2	-66.7
**2020-09**	2	6	1	-83.3
**2020-10**	1	5	3	-40.0
**2020-11**	1	5	1	-80.0
**2020-12**	1	5	1	-80.0
**2021-01**	1	5	1	-80.0
**2021-02**	-	-	0	-
**2021-03**	-	-	1	-
**2021-04**	1	5	3	-40.0
**2021-05**	2	5	3	-40.0
**Health - Other mental disorders**				
**2020-01**	59	73	54	-26.0
**2020-02**	62	76	63	-17.1
**2020-03**	63	77	61	-20.8
**2020-04**	65	79	53	-32.9
**2020-05**	63	77	65	-15.6
**2020-06**	67	81	59	-27.2

© 2022 Koda M et al. *JAMA Network Open.*

	Expected number of monthly suicides (No)	95% upper bound of the expected number of monthly suicides (No)	Observed number of suicides (No)	Percent change (%)
**2020-07**	61	75	70	-6.7
**2020-08**	60	74	73	-1.4
**2020-09**	57	71	68	-4.2
**2020-10**	58	72	70	-2.8
**2020-11**	58	72	60	-16.7
**2020-12**	56	69	48	-30.4
**2021-01**	54	67	54	-19.4
**2021-02**	58	72	55	-23.6
**2021-03**	58	71	60	-15.5
**2021-04**	60	73	63	-13.7
**2021-05**	60	74	51	-31.1
**Health - Physical disability**				
**2020-01**	9	16	10	-37.5
**2020-02**	11	19	8	-57.9
**2020-03**	12	19	11	-42.1
**2020-04**	16	24	11	-54.2
**2020-05**	14	23	10	-56.5
**2020-06**	16	24	9	-62.5
**2020-07**	13	20	21	5.0 *
**2020-08**	16	24	17	-29.2
**2020-09**	13	20	18	-10.0
**2020-10**	14	21	17	-19.0
**2020-11**	13	20	17	-15.0
**2020-12**	13	20	21	5.0 *
**2021-01**	12	21	13	-38.1
**2021-02**	10	17	12	-29.4
**2021-03**	10	17	11	-35.3
**2021-04**	11	17	16	-5.9

© 2022 Koda M et al. *JAMA Network Open.*

	Expected number of monthly suicides (No)	95% upper bound of the expected number of monthly suicides (No)	Observed number of suicides (No)	Percent change (%)
**2021-05**	12	18	14	-22.2
**Health - Others**				
**2020-01**	13	21	9	-57.1
**2020-02**	10	16	9	-43.8
**2020-03**	12	19	13	-31.6
**2020-04**	15	23	17	-26.1
**2020-05**	17	25	20	-20.0
**2020-06**	17	25	11	-56.0
**2020-07**	13	20	19	-5.0
**2020-08**	15	23	13	-43.5
**2020-09**	13	20	12	-40.0
**2020-10**	12	19	13	-31.6
**2020-11**	13	21	14	-33.3
**2020-12**	13	20	14	-30.0
**2021-01**	12	19	12	-36.8
**2021-02**	11	17	14	-17.6
**2021-03**	13	20	7	-65.0
**2021-04**	14	22	9	-59.1
**2021-05**	14	22	13	-40.9
**Economy - Bankruptcy**				
**2020-01**	-	-	1	-
**2020-02**	-	-	1	-
**2020-03**	-	-	2	-
**2020-04**	-	-	0	-
**2020-05**	3	9	2	-77.8
**2020-06**	3	10	1	-90.0
**2020-07**	-	-	0	-
**2020-08**	-	-	1	-

© 2022 Koda M et al. *JAMA Network Open.*

	Expected number of monthly suicides (No)	95% upper bound of the expected number of monthly suicides (No)	Observed number of suicides (No)	Percent change (%)
**2020-09**	-	-	2	-
**2020-10**	2	7	2	-71.4
**2020-11**	1	5	2	-60.0
**2020-12**	1	5	0	-100.0
**2021-01**	-	-	0	-
**2021-02**	-	-	2	-
**2021-03**	-	-	1	-
**2021-04**	-	-	1	-
**2021-05**	1	5	3	-40.0
**Economy - Business slump**				
**2020-01**	28	38	27	-28.9
**2020-02**	29	41	28	-31.7
**2020-03**	30	42	29	-31.0
**2020-04**	28	38	20	-47.4
**2020-05**	24	36	22	-38.9
**2020-06**	23	34	22	-35.3
**2020-07**	23	32	15	-53.1
**2020-08**	25	38	18	-52.6
**2020-09**	26	39	20	-48.7
**2020-10**	31	44	19	-56.8
**2020-11**	28	38	18	-52.6
**2020-12**	28	40	30	-25.0
**2021-01**	28	38	19	-50.0
**2021-02**	26	35	25	-28.6
**2021-03**	25	35	13	-62.9
**2021-04**	21	30	21	-30.0
**2021-05**	21	30	15	-50.0
**Economy - Unemployment**				

© 2022 Koda M et al. *JAMA Network Open.*

	Expected number of monthly suicides (No)	95% upper bound of the expected number of monthly suicides (No)	Observed number of suicides (No)	Percent change (%)
**2020-01**	15	22	16	-27.3
**2020-02**	17	26	19	-26.9
**2020-03**	18	27	25	-7.4
**2020-04**	21	30	22	-26.7
**2020-05**	17	25	17	-32.0
**2020-06**	18	26	13	-50.0
**2020-07**	15	23	20	-13.0
**2020-08**	17	25	13	-48.0
**2020-09**	14	21	30	42.9 *
**2020-10**	18	29	15	-48.3
**2020-11**	15	24	22	-8.3
**2020-12**	17	27	21	-22.2
**2021-01**	18	27	19	-29.6
**2021-02**	20	30	11	-63.3
**2021-03**	19	31	19	-38.7
**2021-04**	21	31	18	-41.9
**2021-05**	18	28	14	-50.0
**Economy - Job failure**				
**2020-01**	12	19	12	-36.8
**2020-02**	14	23	14	-39.1
**2020-03**	16	24	11	-54.2
**2020-04**	15	23	16	-30.4
**2020-05**	14	22	22	0.0
**2020-06**	14	23	13	-43.5
**2020-07**	13	21	5	-76.2
**2020-08**	11	19	10	-47.4
**2020-09**	13	23	18	-21.7
**2020-10**	12	20	8	-60.0

© 2022 Koda M et al. *JAMA Network Open.*

	Expected number of monthly suicides (No)	95% upper bound of the expected number of monthly suicides (No)	Observed number of suicides (No)	Percent change (%)
**2020-11**	10	18	13	-27.8
**2020-12**	11	21	12	-42.9
**2021-01**	12	21	6	-71.4
**2021-02**	11	20	12	-40.0
**2021-03**	13	25	18	-28.0
**2021-04**	17	27	14	-48.1
**2021-05**	16	28	11	-60.7
**Economy - Poverty**				
**2020-01**	66	87	80	-8.0
**2020-02**	81	101	84	-16.8
**2020-03**	84	108	72	-33.3
**2020-04**	80	103	71	-31.1
**2020-05**	68	83	61	-26.5
**2020-06**	63	84	41	-51.2
**2020-07**	55	76	55	-27.6
**2020-08**	62	78	61	-21.8
**2020-09**	60	78	61	-21.8
**2020-10**	59	78	86	10.3 *
**2020-11**	62	85	63	-25.9
**2020-12**	63	84	79	-6.0
**2021-01**	73	97	71	-26.8
**2021-02**	77	96	55	-42.7
**2021-03**	70	92	82	-10.9
**2021-04**	71	93	85	-8.6
**2021-05**	65	89	65	-27.0
**Economy - Multiple debts**				
**2020-01**	53	76	56	-26.3
**2020-02**	63	85	52	-38.8

© 2022 Koda M et al. *JAMA Network Open.*

	Expected number of monthly suicides (No)	95% upper bound of the expected number of monthly suicides (No)	Observed number of suicides (No)	Percent change (%)
**2020-03**	59	84	75	-10.7
**2020-04**	65	88	46	-47.7
**2020-05**	50	70	29	-58.6
**2020-06**	45	68	30	-55.9
**2020-07**	40	63	41	-34.9
**2020-08**	46	65	38	-41.5
**2020-09**	49	69	50	-27.5
**2020-10**	52	77	45	-41.6
**2020-11**	46	60	60	0.0
**2020-12**	51	69	44	-36.2
**2021-01**	48	72	52	-27.8
**2021-02**	59	78	46	-41.0
**2021-03**	55	74	58	-21.6
**2021-04**	52	75	37	-50.7
**2021-05**	35	48	47	-2.1
**Economy - Joint guarantee**				
**2020-01**	-	-	0	-
**2020-02**	-	-	0	-
**2020-03**	-	-	1	-
**2020-04**	-	-	0	-
**2020-05**	-	-	2	-
**2020-06**	-	-	1	-
**2020-07**	-	-	0	-
**2020-08**	-	-	0	-
**2020-09**	-	-	0	-
**2020-10**	-	-	1	-
**2020-11**	-	-	0	-
**2020-12**	-	-	1	-

© 2022 Koda M et al. *JAMA Network Open.*

	Expected number of monthly suicides (No)	95% upper bound of the expected number of monthly suicides (No)	Observed number of suicides (No)	Percent change (%)
**2021-01**	-	-	0	-
**2021-02**	-	-	2	-
**2021-03**	1	6	3	-50.0
**2021-04**	2	7	2	-71.4
**2021-05**	1	5	3	-40.0
**Economy - Other debts**				
**2020-01**	44	56	63	12.5 *
**2020-02**	54	67	39	-41.8
**2020-03**	48	67	49	-26.9
**2020-04**	54	71	33	-53.5
**2020-05**	49	66	31	-53.0
**2020-06**	48	70	23	-67.1
**2020-07**	42	64	33	-48.4
**2020-08**	41	54	31	-42.6
**2020-09**	37	55	51	-7.3
**2020-10**	40	59	46	-22.0
**2020-11**	37	53	32	-39.6
**2020-12**	41	62	40	-35.5
**2021-01**	45	64	40	-37.5
**2021-02**	48	66	29	-56.1
**2021-03**	37	52	53	1.9 *
**2021-04**	42	59	41	-30.5
**2021-05**	32	49	35	-28.6
**Economy - Debt collection trouble**				
**2020-01**	3	9	4	-55.6
**2020-02**	4	10	1	-90.0
**2020-03**	3	8	3	-62.5
**2020-04**	4	11	8	-27.3

© 2022 Koda M et al. *JAMA Network Open.*

	Expected number of monthly suicides (No)	95% upper bound of the expected number of monthly suicides (No)	Observed number of suicides (No)	Percent change (%)
**2020-05**	5	13	3	-76.9
**2020-06**	4	12	4	-66.7
**2020-07**	3	8	3	-62.5
**2020-08**	4	11	1	-90.9
**2020-09**	4	13	4	-69.2
**2020-10**	6	12	8	-33.3
**2020-11**	5	11	4	-63.6
**2020-12**	4	9	3	-66.7
**2021-01**	3	7	4	-42.9
**2021-02**	3	7	4	-42.9
**2021-03**	4	10	4	-60.0
**2021-04**	5	9	2	-77.8
**2021-05**	4	9	3	-66.7
**Economy - Suicide for insurance**				
**2020-01**	4	8	2	-75.0
**2020-02**	3	7	2	-71.4
**2020-03**	2	5	3	-40.0
**2020-04**	2	6	3	-50.0
**2020-05**	3	6	0	-100.0
**2020-06**	2	6	2	-66.7
**2020-07**	2	6	2	-66.7
**2020-08**	2	5	2	-60.0
**2020-09**	2	5	1	-80.0
**2020-10**	2	5	2	-60.0
**2020-11**	2	6	0	-100.0
**2020-12**	2	5	4	-20.0
**2021-01**	3	7	3	-57.1
**2021-02**	2	7	2	-71.4

© 2022 Koda M et al. *JAMA Network Open.*

	Expected number of monthly suicides (No)	95% upper bound of the expected number of monthly suicides (No)	Observed number of suicides (No)	Percent change (%)
**2021-03**	2	7	1	-85.7
**2021-04**	2	6	3	-50.0
**2021-05**	2	6	3	-50.0
**Economy - Others**				
**2020-01**	13	21	23	9.5 *
**2020-02**	17	27	14	-48.1
**2020-03**	17	31	21	-32.3
**2020-04**	19	30	19	-36.7
**2020-05**	19	28	14	-50.0
**2020-06**	19	35	12	-65.7
**2020-07**	19	32	16	-50.0
**2020-08**	19	29	18	-37.9
**2020-09**	14	27	15	-44.4
**2020-10**	10	19	18	-5.3
**2020-11**	10	19	12	-36.8
**2020-12**	13	23	14	-39.1
**2021-01**	16	23	29	26.1 *
**2021-02**	22	31	18	-41.9
**2021-03**	18	28	17	-39.3
**2021-04**	18	26	14	-46.2
**2021-05**	15	22	18	-18.2
**Work - Work failure**				
**2020-01**	24	34	23	-32.4
**2020-02**	25	34	27	-20.6
**2020-03**	26	35	19	-45.7
**2020-04**	23	32	20	-37.5
**2020-05**	25	34	18	-47.1
**2020-06**	24	34	21	-38.2

© 2022 Koda M et al. *JAMA Network Open.*

	Expected number of monthly suicides (No)	95% upper bound of the expected number of monthly suicides (No)	Observed number of suicides (No)	Percent change (%)
**2020-07**	25	35	20	-42.9
**2020-08**	24	33	29	-12.1
**2020-09**	26	35	20	-42.9
**2020-10**	20	28	24	-14.3
**2020-11**	20	29	31	6.9 *
**2020-12**	23	32	22	-31.2
**2021-01**	24	33	21	-36.4
**2021-02**	23	31	21	-32.3
**2021-03**	22	30	24	-20.0
**2021-04**	20	29	30	3.4 *
**2021-05**	22	31	19	-38.7
**Work - Workplace relationships**				
**2020-01**	39	51	44	-13.7
**2020-02**	41	53	20	-62.3
**2020-03**	30	43	51	18.6 *
**2020-04**	36	55	25	-54.5
**2020-05**	28	43	23	-46.5
**2020-06**	32	52	27	-48.1
**2020-07**	33	53	39	-26.4
**2020-08**	37	53	26	-50.9
**2020-09**	30	49	32	-34.7
**2020-10**	31	51	40	-21.6
**2020-11**	32	49	37	-24.5
**2020-12**	36	57	35	-38.6
**2021-01**	33	53	33	-37.7
**2021-02**	37	53	26	-50.9
**2021-03**	31	49	30	-38.8
**2021-04**	32	49	20	-59.2

© 2022 Koda M et al. *JAMA Network Open.*

	Expected number of monthly suicides (No)	95% upper bound of the expected number of monthly suicides (No)	Observed number of suicides (No)	Percent change (%)
**2021-05**	24	33	33	0.0
**Work - Work environment changes**				
**2020-01**	18	27	21	-22.2
**2020-02**	20	28	10	-64.3
**2020-03**	19	29	19	-34.5
**2020-04**	22	32	22	-31.2
**2020-05**	24	33	19	-42.4
**2020-06**	23	32	14	-56.2
**2020-07**	20	30	21	-30.0
**2020-08**	21	30	17	-43.3
**2020-09**	17	25	20	-20.0
**2020-10**	17	26	20	-23.1
**2020-11**	17	24	26	8.3 *
**2020-12**	20	29	16	-44.8
**2021-01**	17	24	17	-29.2
**2021-02**	17	25	23	-8.0
**2021-03**	19	28	19	-32.1
**2021-04**	20	28	18	-35.7
**2021-05**	18	26	19	-26.9
**Work - Work fatigue**				
**2020-01**	48	62	35	-43.5
**2020-02**	45	61	34	-44.3
**2020-03**	41	53	33	-37.7
**2020-04**	42	54	28	-48.1
**2020-05**	38	51	36	-29.4
**2020-06**	36	48	25	-47.9
**2020-07**	33	44	33	-25.0
**2020-08**	39	50	39	-22.0

© 2022 Koda M et al. *JAMA Network Open.*

	Expected number of monthly suicides (No)	95% upper bound of the expected number of monthly suicides (No)	Observed number of suicides (No)	Percent change (%)
**2020-09**	42	54	37	-31.5
**2020-10**	39	51	52	2.0 *
**2020-11**	42	54	51	-5.6
**2020-12**	40	54	38	-29.6
**2021-01**	38	49	39	-20.4
**2021-02**	35	46	28	-39.1
**2021-03**	31	41	55	34.1 *
**2021-04**	38	55	26	-52.7
**2021-05**	29	42	40	-4.8
**Work - Others**				
**2020-01**	22	31	21	-32.3
**2020-02**	26	35	17	-51.4
**2020-03**	24	33	26	-21.2
**2020-04**	29	39	18	-53.8
**2020-05**	24	34	15	-55.9
**2020-06**	23	33	13	-60.6
**2020-07**	20	28	19	-32.1
**2020-08**	20	29	20	-31.0
**2020-09**	19	27	17	-37.0
**2020-10**	18	26	37	42.3 *
**2020-11**	23	35	19	-45.7
**2020-12**	18	29	30	3.4 *
**2021-01**	21	35	28	-20.0
**2021-02**	23	33	24	-27.3
**2021-03**	21	33	27	-18.2
**2021-04**	22	34	13	-61.8
**2021-05**	15	22	17	-22.7
**Relationships - Marriage relationship**				

© 2022 Koda M et al. *JAMA Network Open.*

	Expected number of monthly suicides (No)	95% upper bound of the expected number of monthly suicides (No)	Observed number of suicides (No)	Percent change (%)
**2020-01**	3	7	5	-28.6
**2020-02**	4	9	1	-88.9
**2020-03**	3	7	1	-85.7
**2020-04**	3	7	1	-85.7
**2020-05**	2	6	3	-50.0
**2020-06**	3	7	1	-85.7
**2020-07**	3	7	3	-57.1
**2020-08**	3	8	4	-50.0
**2020-09**	3	6	3	-50.0
**2020-10**	2	6	1	-83.3
**2020-11**	2	6	3	-50.0
**2020-12**	3	7	4	-42.9
**2021-01**	3	8	1	-87.5
**2021-02**	2	6	2	-66.7
**2021-03**	1	4	0	-100.0
**2021-04**	1	5	4	-20.0
**2021-05**	2	6	4	-33.3
**Relationships - Heartbreak**				
**2020-01**	14	22	12	-45.5
**2020-02**	12	19	8	-57.9
**2020-03**	10	17	15	-11.8
**2020-04**	12	19	9	-52.6
**2020-05**	12	18	21	16.7 *
**2020-06**	14	22	3	-86.4
**2020-07**	9	17	20	17.6 *
**2020-08**	13	26	10	-61.5
**2020-09**	12	22	19	-13.6
**2020-10**	15	28	11	-60.7

© 2022 Koda M et al. *JAMA Network Open.*

	Expected number of monthly suicides (No)	95% upper bound of the expected number of monthly suicides (No)	Observed number of suicides (No)	Percent change (%)
**2020-11**	14	27	19	-29.6
**2020-12**	15	28	9	-67.9
**2021-01**	12	24	19	-20.8
**2021-02**	14	28	15	-46.4
**2021-03**	12	24	13	-45.8
**2021-04**	15	28	18	-35.7
**2021-05**	13	26	12	-53.8
**Relationships - Infidelity**				
**2020-01**	7	14	8	-42.9
**2020-02**	8	14	7	-50.0
**2020-03**	6	11	12	9.1 *
**2020-04**	8	15	8	-46.7
**2020-05**	7	13	10	-23.1
**2020-06**	10	16	10	-37.5
**2020-07**	10	16	3	-81.2
**2020-08**	9	16	10	-37.5
**2020-09**	10	16	9	-43.8
**2020-10**	8	15	15	0.0
**2020-11**	9	17	10	-41.2
**2020-12**	7	13	5	-61.5
**2021-01**	6	11	7	-36.4
**2021-02**	9	15	8	-46.7
**2021-03**	9	15	10	-33.3
**2021-04**	10	17	8	-52.9
**2021-05**	9	16	5	-68.8
**Relationships - Other relationship distress**				
**2020-01**	13	21	15	-28.6
**2020-02**	13	21	15	-28.6

	Expected number of monthly suicides (No)	95% upper bound of the expected number of monthly suicides (No)	Observed number of suicides (No)	Percent change (%)
**2020-03**	12	19	11	-42.1
**2020-04**	12	19	7	-63.2
**2020-05**	10	18	8	-55.6
**2020-06**	12	21	5	-76.2
**2020-07**	10	16	12	-25.0
**2020-08**	12	19	17	-10.5
**2020-09**	12	21	9	-57.1
**2020-10**	8	14	18	28.6 *
**2020-11**	12	20	6	-70.0
**2020-12**	11	20	11	-45.0
**2021-01**	14	24	11	-54.2
**2021-02**	13	21	10	-52.4
**2021-03**	11	18	9	-50.0
**2021-04**	9	17	11	-35.3
**2021-05**	8	14	11	-21.4
**Relationships - Others**				
**2020-01**	4	9	2	-77.8
**2020-02**	4	8	1	-87.5
**2020-03**	3	8	3	-62.5
**2020-04**	3	8	3	-62.5
**2020-05**	3	6	1	-83.3
**2020-06**	2	6	3	-50.0
**2020-07**	3	7	6	-14.3
**2020-08**	4	9	4	-55.6
**2020-09**	4	8	4	-50.0
**2020-10**	4	9	4	-55.6
**2020-11**	4	8	3	-62.5
**2020-12**	3	8	1	-87.5

© 2022 Koda M et al. *JAMA Network Open.*

	Expected number of monthly suicides (No)	95% upper bound of the expected number of monthly suicides (No)	Observed number of suicides (No)	Percent change (%)
**2021-01**	2	6	2	-66.7
**2021-02**	2	5	3	-40.0
**2021-03**	2	6	2	-66.7
**2021-04**	2	6	5	-16.7
**2021-05**	3	7	2	-71.4
**School - Admissions**				
**2020-01**	3	8	7	-12.5
**2020-02**	5	10	5	-50.0
**2020-03**	4	9	6	-33.3
**2020-04**	3	9	1	-88.9
**2020-05**	1	4	0	-100.0
**2020-06**	1	5	0	-100.0
**2020-07**	-	-	0	-
**2020-08**	-	-	0	-
**2020-09**	-	-	0	-
**2020-10**	-	-	2	-
**2020-11**	-	-	1	-
**2020-12**	3	11	3	-72.7
**2021-01**	4	12	2	-83.3
**2021-02**	5	12	1	-91.7
**2021-03**	3	10	3	-70.0
**2021-04**	3	8	1	-87.5
**2021-05**	0	4	1	-75.0
**School - Academic path**				
**2020-01**	9	16	6	-62.5
**2020-02**	10	16	6	-62.5
**2020-03**	9	16	8	-50.0
**2020-04**	9	15	6	-60.0

© 2022 Koda M et al. *JAMA Network Open.*

	Expected number of monthly suicides (No)	95% upper bound of the expected number of monthly suicides (No)	Observed number of suicides (No)	Percent change (%)
**2020-05**	6	10	10	0.0
**2020-06**	6	11	6	-45.5
**2020-07**	5	9	7	-22.2
**2020-08**	6	11	9	-18.2
**2020-09**	7	12	6	-50.0
**2020-10**	7	12	9	-25.0
**2020-11**	8	13	9	-30.8
**2020-12**	8	14	2	-85.7
**2021-01**	6	11	8	-27.3
**2021-02**	7	13	7	-46.2
**2021-03**	7	12	6	-50.0
**2021-04**	8	13	6	-53.8
**2021-05**	7	13	7	-46.2
**School - Academic failure**				
**2020-01**	7	13	9	-30.8
**2020-02**	9	15	7	-53.3
**2020-03**	9	15	12	-20.0
**2020-04**	10	16	6	-62.5
**2020-05**	8	14	5	-64.3
**2020-06**	6	11	5	-54.5
**2020-07**	6	12	3	-75.0
**2020-08**	7	12	14	16.7 *
**2020-09**	10	16	10	-37.5
**2020-10**	8	18	8	-55.6
**2020-11**	7	12	13	8.3 *
**2020-12**	8	15	6	-60.0
**2021-01**	7	15	5	-66.7
**2021-02**	8	16	8	-50.0

© 2022 Koda M et al. *JAMA Network Open.*

	Expected number of monthly suicides (No)	95% upper bound of the expected number of monthly suicides (No)	Observed number of suicides (No)	Percent change (%)
**2021-03**	8	15	10	-33.3
**2021-04**	8	17	12	-29.4
**2021-05**	7	15	6	-60.0
**School - Issues with teachers**				
**2020-01**	-	-	1	-
**2020-02**	-	-	1	-
**2020-03**	-	-	0	-
**2020-04**	-	-	0	-
**2020-05**	-	-	1	-
**2020-06**	-	-	0	-
**2020-07**	-	-	0	-
**2020-08**	-	-	0	-
**2020-09**	-	-	0	-
**2020-10**	-	-	0	-
**2020-11**	-	-	0	-
**2020-12**	-	-	0	-
**2021-01**	-	-	0	-
**2021-02**	-	-	0	-
**2021-03**	-	-	1	-
**2021-04**	-	-	1	-
**2021-05**	-	-	0	-
**School - Bullying**				
**2020-01**	-	-	0	-
**2020-02**	-	-	1	-
**2020-03**	-	-	0	-
**2020-04**	-	-	0	-
**2020-05**	-	-	0	-
**2020-06**	-	-	1	-

© 2022 Koda M et al. *JAMA Network Open.*

	Expected number of monthly suicides (No)	95% upper bound of the expected number of monthly suicides (No)	Observed number of suicides (No)	Percent change (%)
**2020-07**	-	-	0	-
**2020-08**	-	-	0	-
**2020-09**	-	-	1	-
**2020-10**	-	-	0	-
**2020-11**	-	-	0	-
**2020-12**	-	-	0	-
**2021-01**	-	-	0	-
**2021-02**	-	-	0	-
**2021-03**	-	-	0	-
**2021-04**	-	-	0	-
**2021-05**	-	-	0	-
**School - Schoolmate trouble**				
**2020-01**	-	-	2	-
**2020-02**	-	-	0	-
**2020-03**	-	-	2	-
**2020-04**	2	7	1	-85.7
**2020-05**	1	5	3	-40.0
**2020-06**	2	6	0	-100.0
**2020-07**	-	-	0	-
**2020-08**	-	-	1	-
**2020-09**	-	-	0	-
**2020-10**	-	-	0	-
**2020-11**	1	5	5	0.0
**2020-12**	2	10	3	-70.0
**2021-01**	1	8	1	-87.5
**2021-02**	1	7	1	-85.7
**2021-03**	1	7	0	-100.0
**2021-04**	-	-	1	-

© 2022 Koda M et al. *JAMA Network Open.*

	Expected number of monthly suicides (No)	95% upper bound of the expected number of monthly suicides (No)	Observed number of suicides (No)	Percent change (%)
**2021-05**	-	-	1	-
**School - Others**				
**2020-01**	3	7	2	-71.4
**2020-02**	2	6	5	-16.7
**2020-03**	4	8	0	-100.0
**2020-04**	2	6	3	-50.0
**2020-05**	4	9	2	-77.8
**2020-06**	3	7	1	-85.7
**2020-07**	2	6	2	-66.7
**2020-08**	3	8	4	-50.0
**2020-09**	4	9	1	-88.9
**2020-10**	3	9	2	-77.8
**2020-11**	3	8	1	-87.5
**2020-12**	-	-	0	-
**2021-01**	2	7	4	-42.9
**2021-02**	3	8	0	-100.0
**2021-03**	2	8	5	-37.5
**2021-04**	2	9	0	-100.0
**2021-05**	2	6	2	-66.7
**Others - Discovery of a crime**				
**2020-01**	14	22	8	-63.6
**2020-02**	13	21	16	-23.8
**2020-03**	15	22	14	-36.4
**2020-04**	16	24	17	-29.2
**2020-05**	17	25	14	-44.0
**2020-06**	15	23	11	-52.2
**2020-07**	13	20	19	-5.0
**2020-08**	14	22	23	4.5 *

© 2022 Koda M et al. *JAMA Network Open.*

	Expected number of monthly suicides (No)	95% upper bound of the expected number of monthly suicides (No)	Observed number of suicides (No)	Percent change (%)
**2020-09**	17	25	9	-64.0
**2020-10**	12	20	13	-35.0
**2020-11**	13	21	21	0.0
**2020-12**	13	24	13	-45.8
**2021-01**	13	21	13	-38.1
**2021-02**	13	21	18	-14.3
**2021-03**	16	26	12	-53.8
**2021-04**	14	23	17	-26.1
**2021-05**	15	24	18	-25.0
**Others - Victim of a crime**				
**2020-01**	-	-	0	-
**2020-02**	-	-	0	-
**2020-03**	-	-	0	-
**2020-04**	-	-	0	-
**2020-05**	-	-	0	-
**2020-06**	-	-	0	-
**2020-07**	-	-	1	-
**2020-08**	-	-	2	-
**2020-09**	-	-	1	-
**2020-10**	-	-	0	-
**2020-11**	-	-	0	-
**2020-12**	-	-	0	-
**2021-01**	-	-	0	-
**2021-02**	-	-	0	-
**2021-03**	-	-	0	-
**2021-04**	-	-	0	-
**2021-05**	-	-	1	-
**Others - Copycat suicide**				

	Expected number of monthly suicides (No)	95% upper bound of the expected number of monthly suicides (No)	Observed number of suicides (No)	Percent change (%)
**2020-01**	4	8	6	-25.0
**2020-02**	4	9	3	-66.7
**2020-03**	4	8	3	-62.5
**2020-04**	3	7	8	14.3 *
**2020-05**	5	10	2	-80.0
**2020-06**	3	7	5	-28.6
**2020-07**	4	8	5	-37.5
**2020-08**	5	10	2	-80.0
**2020-09**	5	11	4	-63.6
**2020-10**	5	11	9	-18.2
**2020-11**	6	12	4	-66.7
**2020-12**	4	10	4	-60.0
**2021-01**	4	9	0	-100.0
**2021-02**	3	9	3	-66.7
**2021-03**	4	11	5	-54.5
**2021-04**	5	11	3	-72.7
**2021-05**	4	11	4	-63.6
**Others - Loneliness**				
**2020-01**	20	28	20	-28.6
**2020-02**	19	27	29	7.4 *
**2020-03**	20	28	27	-3.6
**2020-04**	20	29	22	-24.1
**2020-05**	17	25	21	-16.0
**2020-06**	18	26	18	-30.8
**2020-07**	17	25	19	-24.0
**2020-08**	17	25	21	-16.0
**2020-09**	16	24	30	25.0 *
**2020-10**	19	29	20	-31.0

	Expected number of monthly suicides (No)	95% upper bound of the expected number of monthly suicides (No)	Observed number of suicides (No)	Percent change (%)
**2020-11**	19	27	26	-3.7
**2020-12**	22	31	25	-19.4
**2021-01**	24	33	26	-21.2
**2021-02**	26	35	17	-51.4
**2021-03**	24	33	23	-30.3
**2021-04**	23	32	25	-21.9
**2021-05**	22	30	16	-46.7
**Others - Neighborhood trouble**				
**2020-01**	1	5	2	-60.0
**2020-02**	2	9	1	-88.9
**2020-03**	3	9	2	-77.8
**2020-04**	3	8	7	-12.5
**2020-05**	4	11	4	-63.6
**2020-06**	2	7	1	-85.7
**2020-07**	1	3	1	-66.7
**2020-08**	1	5	2	-60.0
**2020-09**	2	7	1	-85.7
**2020-10**	3	6	3	-50.0
**2020-11**	2	6	0	-100.0
**2020-12**	2	6	2	-66.7
**2021-01**	1	5	3	-40.0
**2021-02**	2	6	1	-83.3
**2021-03**	3	9	1	-88.9
**2021-04**	3	9	7	-22.2
**2021-05**	5	11	1	-90.9
**Others - Others**				
**2020-01**	25	36	22	-38.9
**2020-02**	28	37	16	-56.8

© 2022 Koda M et al. *JAMA Network Open.*

	Expected number of monthly suicides (No)	95% upper bound of the expected number of monthly suicides (No)	Observed number of suicides (No)	Percent change (%)
**2020-03**	29	42	26	-38.1
**2020-04**	30	40	21	-47.5
**2020-05**	29	39	19	-51.3
**2020-06**	24	34	20	-41.2
**2020-07**	24	33	22	-33.3
**2020-08**	21	29	37	27.6 *
**2020-09**	26	36	24	-33.3
**2020-10**	22	31	21	-32.3
**2020-11**	21	30	25	-16.7
**2020-12**	22	31	22	-29.0
**2021-01**	20	28	25	-10.7
**2021-02**	22	31	22	-29.0
**2021-03**	21	30	27	-10.0
**2021-04**	23	32	19	-40.6
**2021-05**	20	28	28	0.0

Percent change was defined as the difference between the observed number of suicides for a month and the 95% upper bound of the expected number of suicides for that month divided by the threshold. * Indicates a month with the observed number of suicides exceeding the 95% upper bound of the expected number of suicides for that month. - Indicates a month for which we were unable to calculate the percent owing to a small sample.

eTable 3. **Expected and Observed Number of Monthly Suicides and Percentage Change From January 2020 to May 2021 by 52 Subcategories Among Women**

	Expected number of monthly suicides (No)	95% upper bound of the expected number of monthly suicides (No)	Observed number of suicides (No)	Percent changes (%)
**Family – Parent–child problems**				
**2020-01**	14	22	18	-18.2
**2020-02**	15	23	15	-34.8
**2020-03**	15	22	23	4.5 *
**2020-04**	16	24	12	-50.0
**2020-05**	14	22	17	-22.7
**2020-06**	17	26	14	-46.2
**2020-07**	18	27	25	-7.4
**2020-08**	21	30	19	-36.7
**2020-09**	17	27	27	0.0
**2020-10**	19	30	24	-20.0
**2020-11**	17	26	15	-42.3
**2020-12**	17	26	15	-42.3
**2021-01**	15	24	25	4.2 *
**2021-02**	20	29	16	-44.8
**2021-03**	17	26	20	-23.1
**2021-04**	18	28	15	-46.4
**2021-05**	15	22	18	-18.2
**Family - Marital discord**				
**2020-01**	16	23	9	-60.9
**2020-02**	14	23	13	-43.5
**2020-03**	16	25	12	-52.0
**2020-04**	17	26	12	-53.8
**2020-05**	16	24	9	-62.5
**2020-06**	14	23	24	4.3 *

© 2022 Koda M et al. *JAMA Network Open.*

	Expected number of monthly suicides (No)	95% upper bound of the expected number of monthly suicides (No)	Observed number of suicides (No)	Percent changes (%)
**2020-07**	18	26	18	-30.8
**2020-08**	16	26	18	-30.8
**2020-09**	17	26	21	-19.2
**2020-10**	15	23	32	39.1 *
**2020-11**	19	34	33	-2.9
**2020-12**	18	35	21	-40.0
**2021-01**	16	24	24	0.0
**2021-02**	15	27	19	-29.6
**2021-03**	14	24	16	-33.3
**2021-04**	12	19	16	-15.8
**2021-05**	15	25	16	-36.0
**Family - Other family discords**				
**2020-01**	9	17	10	-41.2
**2020-02**	10	17	8	-52.9
**2020-03**	10	17	9	-47.1
**2020-04**	10	17	8	-52.9
**2020-05**	10	17	4	-76.5
**2020-06**	6	12	9	-25.0
**2020-07**	9	15	11	-26.7
**2020-08**	9	15	12	-20.0
**2020-09**	9	16	8	-50.0
**2020-10**	8	14	15	7.1 *
**2020-11**	10	16	17	6.2 *
**2020-12**	11	19	19	0.0
**2021-01**	11	20	7	-65.0
**2021-02**	9	14	13	-7.1
**2021-03**	10	17	13	-23.5
**2021-04**	9	16	8	-50.0

© 2022 Koda M et al. *JAMA Network Open.*

	Expected number of monthly suicides (No)	95% upper bound of the expected number of monthly suicides (No)	Observed number of suicides (No)	Percent changes (%)
**2021-05**	7	13	12	-7.7
**Family - Death of a family member**				
**2020-01**	11	20	19	-5.0
**2020-02**	15	24	12	-50.0
**2020-03**	14	23	18	-21.7
**2020-04**	17	25	12	-52.0
**2020-05**	15	23	20	-13.0
**2020-06**	17	26	16	-38.5
**2020-07**	17	25	21	-16.0
**2020-08**	18	27	11	-59.3
**2020-09**	15	23	13	-43.5
**2020-10**	15	22	22	0.0
**2020-11**	17	26	17	-34.6
**2020-12**	15	23	12	-47.8
**2021-01**	14	22	10	-54.5
**2021-02**	15	24	17	-29.2
**2021-03**	15	22	13	-40.9
**2021-04**	16	24	10	-58.3
**2021-05**	15	24	12	-50.0
**Family - Pessimism about the future of the family**				
**2020-01**	13	20	8	-60.0
**2020-02**	12	19	16	-15.8
**2020-03**	16	25	17	-32.0
**2020-04**	16	24	6	-75.0
**2020-05**	14	24	5	-79.2
**2020-06**	12	22	16	-27.3
**2020-07**	14	24	12	-50.0
**2020-08**	13	23	10	-56.5

© 2022 Koda M et al. *JAMA Network Open.*

	Expected number of monthly suicides (No)	95% upper bound of the expected number of monthly suicides (No)	Observed number of suicides (No)	Percent changes (%)
**2020-09**	13	24	19	-20.8
**2020-10**	16	28	22	-21.4
**2020-11**	16	29	16	-44.8
**2020-12**	14	26	18	-30.8
**2021-01**	13	24	21	-12.5
**2021-02**	16	27	15	-44.4
**2021-03**	14	24	22	-8.3
**2021-04**	12	24	14	-41.7
**2021-05**	10	19	15	-21.1
**Family - Abuse from family**				
**2020-01**	1	4	4	0.0
**2020-02**	3	7	2	-71.4
**2020-03**	4	8	4	-50.0
**2020-04**	4	9	2	-77.8
**2020-05**	3	7	5	-28.6
**2020-06**	3	7	3	-57.1
**2020-07**	3	6	2	-66.7
**2020-08**	4	8	7	-12.5
**2020-09**	5	10	8	-20.0
**2020-10**	5	11	4	-63.6
**2020-11**	3	7	2	-71.4
**2020-12**	3	6	4	-33.3
**2021-01**	3	8	4	-50.0
**2021-02**	4	8	2	-75.0
**2021-03**	3	6	4	-33.3
**2021-04**	4	9	2	-77.8
**2021-05**	3	7	1	-85.7
**Family - Child-rearing problems**				

© 2022 Koda M et al. *JAMA Network Open.*

	Expected number of monthly suicides (No)	95% upper bound of the expected number of monthly suicides (No)	Observed number of suicides (No)	Percent changes (%)
**2020-01**	9	15	9	-40.0
**2020-02**	10	16	6	-62.5
**2020-03**	8	16	7	-56.2
**2020-04**	7	13	5	-61.5
**2020-05**	8	15	4	-73.3
**2020-06**	9	18	5	-72.2
**2020-07**	10	17	13	-23.5
**2020-08**	10	17	6	-64.7
**2020-09**	6	14	13	-7.1
**2020-10**	6	15	21	40.0 *
**2020-11**	8	21	10	-52.4
**2020-12**	7	16	15	-6.2
**2021-01**	9	18	12	-33.3
**2021-02**	9	15	10	-33.3
**2021-03**	7	15	9	-40.0
**2021-04**	6	13	3	-76.9
**2021-05**	4	9	11	22.2 *
**Family - Abuse**				
**2020-01**	-	-	0	-
**2020-02**	-	-	0	-
**2020-03**	-	-	0	-
**2020-04**	-	-	0	-
**2020-05**	-	-	0	-
**2020-06**	-	-	0	-
**2020-07**	-	-	0	-
**2020-08**	-	-	0	-
**2020-09**	-	-	1	-
**2020-10**	-	-	0	-

© 2022 Koda M et al. *JAMA Network Open.*

	Expected number of monthly suicides (No)	95% upper bound of the expected number of monthly suicides (No)	Observed number of suicides (No)	Percent changes (%)
**2020-11**	-	-	1	-
**2020-12**	-	-	0	-
**2021-01**	-	-	0	-
**2021-02**	-	-	0	-
**2021-03**	-	-	0	-
**2021-04**	-	-	0	-
**2021-05**	-	-	0	-
**Family - Caregiving fatigue**				
**2020-01**	9	15	4	-73.3
**2020-02**	8	14	6	-57.1
**2020-03**	8	14	12	-14.3
**2020-04**	10	16	4	-75.0
**2020-05**	8	15	6	-60.0
**2020-06**	8	14	5	-64.3
**2020-07**	6	12	5	-58.3
**2020-08**	6	11	6	-45.5
**2020-09**	6	12	6	-50.0
**2020-10**	7	13	6	-53.8
**2020-11**	7	12	4	-66.7
**2020-12**	6	11	7	-36.4
**2021-01**	6	11	8	-27.3
**2021-02**	8	13	4	-69.2
**2021-03**	7	12	15	25.0 *
**2021-04**	9	16	6	-62.5
**2021-05**	5	10	6	-40.0
**Family - Others**				
**2020-01**	8	14	9	-35.7
**2020-02**	10	16	8	-50.0

© 2022 Koda M et al. *JAMA Network Open.*

	Expected number of monthly suicides (No)	95% upper bound of the expected number of monthly suicides (No)	Observed number of suicides (No)	Percent changes (%)
**2020-03**	8	13	5	-61.5
**2020-04**	7	13	9	-30.8
**2020-05**	8	13	10	-23.1
**2020-06**	8	13	11	-15.4
**2020-07**	9	14	11	-21.4
**2020-08**	9	15	10	-33.3
**2020-09**	9	15	12	-20.0
**2020-10**	9	15	19	26.7 *
**2020-11**	10	18	13	-27.8
**2020-12**	9	15	7	-53.3
**2021-01**	7	13	9	-30.8
**2021-02**	8	13	11	-15.4
**2021-03**	8	14	5	-64.3
**2021-04**	7	13	5	-61.5
**2021-05**	9	15	9	-40.0
**Health - Physical illness**				
**2020-01**	70	89	62	-30.3
**2020-02**	75	97	61	-37.1
**2020-03**	72	93	63	-32.3
**2020-04**	77	94	81	-13.8
**2020-05**	82	102	75	-26.5
**2020-06**	86	109	96	-11.9
**2020-07**	87	104	120	15.4 *
**2020-08**	96	123	103	-16.3
**2020-09**	88	120	85	-29.2
**2020-10**	87	103	124	20.4 *
**2020-11**	87	121	78	-35.5
**2020-12**	72	101	77	-23.8

© 2022 Koda M et al. *JAMA Network Open.*

	Expected number of monthly suicides (No)	95% upper bound of the expected number of monthly suicides (No)	Observed number of suicides (No)	Percent changes (%)
**2021-01**	64	87	64	-26.4
**2021-02**	63	86	51	-40.7
**2021-03**	64	89	74	-16.9
**2021-04**	73	98	92	-6.1
**2021-05**	86	117	81	-30.8
**Health - Depression**				
**2020-01**	141	170	157	-7.6
**2020-02**	145	175	133	-24.0
**2020-03**	142	163	158	-3.1
**2020-04**	154	184	125	-32.1
**2020-05**	148	184	143	-22.3
**2020-06**	161	184	143	-22.3
**2020-07**	152	185	213	15.1 *
**2020-08**	163	211	208	-1.4
**2020-09**	149	205	185	-9.8
**2020-10**	152	196	263	34.2 *
**2020-11**	152	188	176	-6.4
**2020-12**	160	209	165	-21.1
**2021-01**	150	200	158	-21.0
**2021-02**	152	195	143	-26.7
**2021-03**	140	184	179	-2.7
**2021-04**	151	204	139	-31.9
**2021-05**	138	180	154	-14.4
**Health - Schizophrenia**				
**2020-01**	37	49	35	-28.6
**2020-02**	35	46	29	-37.0
**2020-03**	35	46	38	-17.4
**2020-04**	36	47	44	-6.4

© 2022 Koda M et al. *JAMA Network Open.*

	Expected number of monthly suicides (No)	95% upper bound of the expected number of monthly suicides (No)	Observed number of suicides (No)	Percent changes (%)
**2020-05**	39	50	34	-32.0
**2020-06**	33	43	30	-30.2
**2020-07**	31	42	34	-19.0
**2020-08**	31	41	39	-4.9
**2020-09**	34	44	40	-9.1
**2020-10**	35	46	58	26.1 *
**2020-11**	42	56	39	-30.4
**2020-12**	36	47	28	-40.4
**2021-01**	32	42	33	-21.4
**2021-02**	34	48	37	-22.9
**2021-03**	37	49	33	-32.7
**2021-04**	37	49	25	-49.0
**2021-05**	33	49	23	-53.1
**Health - Alcoholism**				
**2020-01**	1	4	3	-25.0
**2020-02**	3	6	1	-83.3
**2020-03**	2	6	2	-66.7
**2020-04**	3	7	3	-57.1
**2020-05**	4	9	2	-77.8
**2020-06**	4	10	2	-80.0
**2020-07**	5	10	4	-60.0
**2020-08**	5	10	2	-80.0
**2020-09**	4	9	8	-11.1
**2020-10**	5	11	16	45.5 *
**2020-11**	6	17	2	-88.2
**2020-12**	3	9	3	-66.7
**2021-01**	2	7	2	-71.4
**2021-02**	2	8	2	-75.0

© 2022 Koda M et al. *JAMA Network Open.*

	Expected number of monthly suicides (No)	95% upper bound of the expected number of monthly suicides (No)	Observed number of suicides (No)	Percent changes (%)
**2021-03**	2	6	3	-50.0
**2021-04**	3	7	2	-71.4
**2021-05**	2	9	0	-100.0
**Health - Drug and substance abuse**				
**2020-01**	1	6	0	-100.0
**2020-02**	1	4	1	-75.0
**2020-03**	-	-	1	-
**2020-04**	-	-	0	-
**2020-05**	-	-	1	-
**2020-06**	2	6	4	-33.3
**2020-07**	1	6	0	-100.0
**2020-08**	1	5	0	-100.0
**2020-09**	1	6	1	-83.3
**2020-10**	2	7	4	-42.9
**2020-11**	3	8	5	-37.5
**2020-12**	2	9	1	-88.9
**2021-01**	1	6	1	-83.3
**2021-02**	1	5	0	-100.0
**2021-03**	-	-	1	-
**2021-04**	-	-	0	-
**2021-05**	-	-	0	-
**Health - Other mental disorders**				
**2020-01**	50	70	43	-38.6
**2020-02**	45	63	46	-27.0
**2020-03**	47	65	54	-16.9
**2020-04**	50	69	47	-31.9
**2020-05**	49	67	65	-3.0
**2020-06**	55	74	62	-16.2

© 2022 Koda M et al. *JAMA Network Open.*

	Expected number of monthly suicides (No)	95% upper bound of the expected number of monthly suicides (No)	Observed number of suicides (No)	Percent changes (%)
**2020-07**	52	73	56	-23.3
**2020-08**	54	74	67	-9.5
**2020-09**	50	70	83	18.6 *
**2020-10**	62	85	79	-7.1
**2020-11**	60	84	62	-26.2
**2020-12**	56	71	46	-35.2
**2021-01**	47	59	55	-6.8
**2021-02**	50	69	49	-29.0
**2021-03**	49	61	52	-14.8
**2021-04**	54	68	68	0.0
**2021-05**	61	81	41	-49.4
**Health - Physical disability**				
**2020-01**	9	16	7	-56.2
**2020-02**	8	14	3	-78.6
**2020-03**	7	13	5	-61.5
**2020-04**	6	11	3	-72.7
**2020-05**	7	13	10	-23.1
**2020-06**	8	14	9	-35.7
**2020-07**	8	16	6	-62.5
**2020-08**	6	11	6	-45.5
**2020-09**	5	10	3	-70.0
**2020-10**	6	12	12	0.0
**2020-11**	9	15	12	-20.0
**2020-12**	9	16	7	-56.2
**2021-01**	6	12	6	-50.0
**2021-02**	5	10	4	-60.0
**2021-03**	4	8	8	0.0
**2021-04**	7	13	8	-38.5

© 2022 Koda M et al. *JAMA Network Open.*

	Expected number of monthly suicides (No)	95% upper bound of the expected number of monthly suicides (No)	Observed number of suicides (No)	Percent changes (%)
**2021-05**	8	13	5	-61.5
**Health - Others**				
**2020-01**	7	12	9	-25.0
**2020-02**	9	15	7	-53.3
**2020-03**	9	14	7	-50.0
**2020-04**	8	14	10	-28.6
**2020-05**	8	14	12	-14.3
**2020-06**	10	16	11	-31.2
**2020-07**	9	15	2	-86.7
**2020-08**	7	12	12	0.0
**2020-09**	8	16	12	-25.0
**2020-10**	9	17	21	23.5 *
**2020-11**	11	22	8	-63.6
**2020-12**	8	15	7	-53.3
**2021-01**	7	15	6	-60.0
**2021-02**	7	16	8	-50.0
**2021-03**	8	15	3	-80.0
**2021-04**	8	17	8	-52.9
**2021-05**	10	21	7	-66.7
**Economy - Bankruptcy**				
**2020-01**	-	-	0	-
**2020-02**	-	-	0	-
**2020-03**	-	-	0	-
**2020-04**	-	-	0	-
**2020-05**	-	-	1	-
**2020-06**	-	-	0	-
**2020-07**	-	-	0	-
**2020-08**	-	-	0	-

© 2022 Koda M et al. *JAMA Network Open.*

	Expected number of monthly suicides (No)	95% upper bound of the expected number of monthly suicides (No)	Observed number of suicides (No)	Percent changes (%)
**2020-09**	-	-	1	-
**2020-10**	-	-	1	-
**2020-11**	-	-	0	-
**2020-12**	-	-	0	-
**2021-01**	-	-	0	-
**2021-02**	-	-	0	-
**2021-03**	-	-	0	-
**2021-04**	-	-	0	-
**2021-05**	-	-	1	-
**Economy - Business slump**				
**2020-01**	1	7	2	-71.4
**2020-02**	2	10	0	-100.0
**2020-03**	2	10	3	-70.0
**2020-04**	3	10	1	-90.0
**2020-05**	2	7	1	-85.7
**2020-06**	2	7	1	-85.7
**2020-07**	1	5	6	20.0 *
**2020-08**	2	8	1	-87.5
**2020-09**	1	6	1	-83.3
**2020-10**	2	8	1	-87.5
**2020-11**	-	-	1	-
**2020-12**	-	-	1	-
**2021-01**	-	-	1	-
**2021-02**	2	5	3	-40.0
**2021-03**	2	6	2	-66.7
**2021-04**	2	6	2	-66.7
**2021-05**	1	4	1	-75.0
**Economy - Unemployment**				

© 2022 Koda M et al. *JAMA Network Open.*

	Expected number of monthly suicides (No)	95% upper bound of the expected number of monthly suicides (No)	Observed number of suicides (No)	Percent changes (%)
**2020-01**	2	7	2	-71.4
**2020-02**	1	5	1	-80.0
**2020-03**	1	5	2	-60.0
**2020-04**	2	5	3	-40.0
**2020-05**	3	6	4	-33.3
**2020-06**	3	7	0	-100.0
**2020-07**	2	6	1	-83.3
**2020-08**	2	7	3	-57.1
**2020-09**	2	6	2	-66.7
**2020-10**	2	6	2	-66.7
**2020-11**	2	6	5	-16.7
**2020-12**	3	8	8	0.0
**2021-01**	4	10	1	-90.0
**2021-02**	2	5	1	-80.0
**2021-03**	2	6	4	-33.3
**2021-04**	3	10	3	-70.0
**2021-05**	3	7	2	-71.4
**Economy - Job failure**				
**2020-01**	2	6	1	-83.3
**2020-02**	2	5	3	-40.0
**2020-03**	3	7	3	-57.1
**2020-04**	3	7	4	-42.9
**2020-05**	3	8	1	-87.5
**2020-06**	2	6	3	-50.0
**2020-07**	3	7	4	-42.9
**2020-08**	2	6	4	-33.3
**2020-09**	2	6	2	-66.7
**2020-10**	2	5	3	-40.0

© 2022 Koda M et al. *JAMA Network Open.*

	Expected number of monthly suicides (No)	95% upper bound of the expected number of monthly suicides (No)	Observed number of suicides (No)	Percent changes (%)
**2020-11**	2	6	4	-33.3
**2020-12**	3	6	1	-83.3
**2021-01**	2	5	1	-80.0
**2021-02**	2	6	5	-16.7
**2021-03**	4	9	7	-22.2
**2021-04**	4	9	2	-77.8
**2021-05**	2	7	0	-100.0
**Economy - Poverty**				
**2020-01**	10	17	18	5.9 *
**2020-02**	15	26	13	-50.0
**2020-03**	14	25	14	-44.0
**2020-04**	17	25	15	-40.0
**2020-05**	14	21	16	-23.8
**2020-06**	14	21	8	-61.9
**2020-07**	9	15	16	6.7 *
**2020-08**	12	19	13	-31.6
**2020-09**	12	19	8	-57.9
**2020-10**	12	19	24	26.3 *
**2020-11**	16	26	12	-53.8
**2020-12**	14	24	15	-37.5
**2021-01**	14	24	10	-58.3
**2021-02**	14	23	13	-43.5
**2021-03**	14	23	16	-30.4
**2021-04**	15	25	16	-36.0
**2021-05**	14	22	21	-4.5
**Economy - Multiple debts**				
**2020-01**	5	10	2	-80.0
**2020-02**	4	8	2	-75.0

© 2022 Koda M et al. *JAMA Network Open.*

	Expected number of monthly suicides (No)	95% upper bound of the expected number of monthly suicides (No)	Observed number of suicides (No)	Percent changes (%)
**2020-03**	4	8	5	-37.5
**2020-04**	3	8	2	-75.0
**2020-05**	3	6	2	-66.7
**2020-06**	3	8	2	-75.0
**2020-07**	4	9	3	-66.7
**2020-08**	4	9	3	-66.7
**2020-09**	4	8	5	-37.5
**2020-10**	4	9	5	-44.4
**2020-11**	5	10	1	-90.0
**2020-12**	3	8	5	-37.5
**2021-01**	3	8	3	-62.5
**2021-02**	3	7	4	-42.9
**2021-03**	3	8	2	-75.0
**2021-04**	3	7	4	-42.9
**2021-05**	3	6	7	16.7 *
**Economy - Joint guarantee**				
**2020-01**	-	-	0	-
**2020-02**	-	-	0	-
**2020-03**	-	-	1	-
**2020-04**	-	-	0	-
**2020-05**	-	-	0	-
**2020-06**	-	-	0	-
**2020-07**	-	-	0	-
**2020-08**	-	-	0	-
**2020-09**	-	-	1	-
**2020-10**	-	-	0	-
**2020-11**	-	-	0	-
**2020-12**	-	-	0	-

© 2022 Koda M et al. *JAMA Network Open.*

	Expected number of monthly suicides (No)	95% upper bound of the expected number of monthly suicides (No)	Observed number of suicides (No)	Percent changes (%)
**2021-01**	-	-	0	-
**2021-02**	-	-	0	-
**2021-03**	-	-	1	-
**2021-04**	-	-	0	-
**2021-05**	-	-	0	-
**Economy - Other debts**				
**2020-01**	6	11	8	-27.3
**2020-02**	6	13	3	-76.9
**2020-03**	6	13	6	-53.8
**2020-04**	8	13	4	-69.2
**2020-05**	6	12	1	-91.7
**2020-06**	4	10	3	-70.0
**2020-07**	2	6	3	-50.0
**2020-08**	3	7	2	-71.4
**2020-09**	2	6	5	-16.7
**2020-10**	5	10	7	-30.0
**2020-11**	6	12	5	-58.3
**2020-12**	7	13	7	-46.2
**2021-01**	6	12	7	-41.7
**2021-02**	6	11	4	-63.6
**2021-03**	4	9	4	-55.6
**2021-04**	4	8	5	-37.5
**2021-05**	3	7	5	-28.6
**Economy - Debt collection trouble**				
**2020-01**	-	-	0	-
**2020-02**	-	-	0	-
**2020-03**	-	-	0	-
**2020-04**	-	-	2	-

© 2022 Koda M et al. *JAMA Network Open.*

	Expected number of monthly suicides (No)	95% upper bound of the expected number of monthly suicides (No)	Observed number of suicides (No)	Percent changes (%)
**2020-05**	-	-	0	-
**2020-06**	-	-	1	-
**2020-07**	-	-	1	-
**2020-08**	-	-	0	-
**2020-09**	-	-	1	-
**2020-10**	-	-	0	-
**2020-11**	-	-	3	-
**2020-12**	1	7	1	-85.7
**2021-01**	1	5	1	-80.0
**2021-02**	0	6	1	-83.3
**2021-03**	-	-	0	-
**2021-04**	-	-	0	-
**2021-05**	-	-	0	-
**Economy - Suicide for insurance**				
**2020-01**	-	-	0	-
**2020-02**	-	-	0	-
**2020-03**	-	-	0	-
**2020-04**	-	-	1	-
**2020-05**	-	-	0	-
**2020-06**	-	-	0	-
**2020-07**	-	-	0	-
**2020-08**	-	-	2	-
**2020-09**	-	-	1	-
**2020-10**	-	-	0	-
**2020-11**	-	-	0	-
**2020-12**	-	-	0	-
**2021-01**	-	-	1	-
**2021-02**	-	-	0	-

© 2022 Koda M et al. *JAMA Network Open.*

	Expected number of monthly suicides (No)	95% upper bound of the expected number of monthly suicides (No)	Observed number of suicides (No)	Percent changes (%)
**2021-03**	-	-	0	-
**2021-04**	-	-	0	-
**2021-05**	-	-	1	-
**Economy - Others**				
**2020-01**	3	7	4	-42.9
**2020-02**	5	9	2	-77.8
**2020-03**	5	11	3	-72.7
**2020-04**	5	10	5	-50.0
**2020-05**	5	11	4	-63.6
**2020-06**	5	10	2	-80.0
**2020-07**	4	9	1	-88.9
**2020-08**	5	11	7	-36.4
**2020-09**	6	12	8	-33.3
**2020-10**	7	13	9	-30.8
**2020-11**	5	12	8	-33.3
**2020-12**	5	10	6	-40.0
**2021-01**	3	8	6	-25.0
**2021-02**	4	9	5	-44.4
**2021-03**	4	8	6	-25.0
**2021-04**	4	10	1	-90.0
**2021-05**	3	7	9	28.6 *
**Work - Work failure**				
**2020-01**	1	5	6	20.0 *
**2020-02**	2	9	3	-66.7
**2020-03**	2	7	1	-85.7
**2020-04**	3	7	1	-85.7
**2020-05**	2	7	1	-85.7
**2020-06**	2	7	2	-71.4

© 2022 Koda M et al. *JAMA Network Open.*

	Expected number of monthly suicides (No)	95% upper bound of the expected number of monthly suicides (No)	Observed number of suicides (No)	Percent changes (%)
**2020-07**	2	5	7	40.0 *
**2020-08**	4	9	5	-44.4
**2020-09**	3	9	2	-77.8
**2020-10**	3	7	2	-71.4
**2020-11**	2	6	5	-16.7
**2020-12**	4	10	3	-70.0
**2021-01**	4	8	3	-62.5
**2021-02**	3	8	3	-62.5
**2021-03**	2	6	2	-66.7
**2021-04**	1	4	0	-100.0
**2021-05**	1	4	5	25.0 *
**Work - Workplace relationships**				
**2020-01**	7	13	10	-23.1
**2020-02**	7	13	4	-69.2
**2020-03**	6	11	13	18.2 *
**2020-04**	9	16	12	-25.0
**2020-05**	9	15	8	-46.7
**2020-06**	7	13	6	-53.8
**2020-07**	6	11	12	9.1 *
**2020-08**	7	14	8	-42.9
**2020-09**	7	12	13	8.3 *
**2020-10**	9	16	17	6.2 *
**2020-11**	11	19	13	-31.6
**2020-12**	10	18	7	-61.1
**2021-01**	7	13	6	-53.8
**2021-02**	8	16	4	-75.0
**2021-03**	8	17	12	-29.4
**2021-04**	11	18	9	-50.0

© 2022 Koda M et al. *JAMA Network Open.*

	Expected number of monthly suicides (No)	95% upper bound of the expected number of monthly suicides (No)	Observed number of suicides (No)	Percent changes (%)
**2021-05**	9	16	7	-56.2
**Work - Work environment changes**				
**2020-01**	1	5	5	0.0
**2020-02**	3	7	1	-85.7
**2020-03**	2	5	3	-40.0
**2020-04**	2	6	2	-66.7
**2020-05**	2	7	3	-57.1
**2020-06**	3	8	4	-50.0
**2020-07**	4	9	9	0.0
**2020-08**	4	11	3	-72.7
**2020-09**	2	7	5	-28.6
**2020-10**	2	8	8	0.0
**2020-11**	3	10	4	-60.0
**2020-12**	3	8	1	-87.5
**2021-01**	2	7	3	-57.1
**2021-02**	3	8	1	-87.5
**2021-03**	2	5	4	-20.0
**2021-04**	3	7	7	0.0
**2021-05**	4	10	2	-80.0
**Work - Work fatigue**				
**2020-01**	3	8	3	-62.5
**2020-02**	4	9	3	-66.7
**2020-03**	5	12	4	-66.7
**2020-04**	5	11	1	-90.9
**2020-05**	5	12	4	-66.7
**2020-06**	5	13	3	-76.9
**2020-07**	5	12	5	-58.3
**2020-08**	4	10	9	-10.0

© 2022 Koda M et al. *JAMA Network Open.*

	Expected number of monthly suicides (No)	95% upper bound of the expected number of monthly suicides (No)	Observed number of suicides (No)	Percent changes (%)
**2020-09**	7	16	14	-12.5
**2020-10**	9	18	10	-44.4
**2020-11**	8	15	8	-46.7
**2020-12**	6	11	5	-54.5
**2021-01**	3	8	5	-37.5
**2021-02**	4	8	3	-62.5
**2021-03**	3	6	14	133.3 *
**2021-04**	6	15	8	-46.7
**2021-05**	4	12	8	-33.3
**Work - Others**				
**2020-01**	4	8	3	-62.5
**2020-02**	5	9	1	-88.9
**2020-03**	4	8	1	-87.5
**2020-04**	3	7	3	-57.1
**2020-05**	3	7	3	-57.1
**2020-06**	3	7	6	-14.3
**2020-07**	4	8	6	-25.0
**2020-08**	4	8	5	-37.5
**2020-09**	4	8	4	-50.0
**2020-10**	4	8	4	-50.0
**2020-11**	4	8	6	-25.0
**2020-12**	4	9	7	-22.2
**2021-01**	3	8	5	-37.5
**2021-02**	3	6	2	-66.7
**2021-03**	2	5	2	-60.0
**2021-04**	2	6	3	-50.0
**2021-05**	4	8	4	-50.0
**Relationships - Marriage**				

© 2022 Koda M et al. *JAMA Network Open.*

	Expected number of monthly suicides (No)	95% upper bound of the expected number of monthly suicides (No)	Observed number of suicides (No)	Percent changes (%)
**2020-01**	-	-	0	-
**2020-02**	2	7	1	-85.7
**2020-03**	-	-	1	-
**2020-04**	-	-	1	-
**2020-05**	-	-	1	-
**2020-06**	-	-	1	-
**2020-07**	2	6	2	-66.7
**2020-08**	-	-	0	-
**2020-09**	1	4	2	-50.0
**2020-10**	2	5	4	-20.0
**2020-11**	2	6	2	-66.7
**2020-12**	2	5	3	-40.0
**2021-01**	2	5	1	-80.0
**2021-02**	1	3	2	-33.3
**2021-03**	1	4	3	-25.0
**2021-04**	2	5	3	-40.0
**2021-05**	1	5	2	-60.0
**Relationships - Heartbreak**				
**2020-01**	7	16	7	-56.2
**2020-02**	5	12	9	-25.0
**2020-03**	5	10	9	-10.0
**2020-04**	7	14	5	-64.3
**2020-05**	7	14	4	-71.4
**2020-06**	8	13	2	-84.6
**2020-07**	7	13	10	-23.1
**2020-08**	8	14	11	-21.4
**2020-09**	9	16	6	-62.5
**2020-10**	8	15	12	-20.0

© 2022 Koda M et al. *JAMA Network Open.*

	Expected number of monthly suicides (No)	95% upper bound of the expected number of monthly suicides (No)	Observed number of suicides (No)	Percent changes (%)
**2020-11**	10	16	7	-56.2
**2020-12**	8	15	9	-40.0
**2021-01**	8	16	7	-56.2
**2021-02**	8	14	6	-57.1
**2021-03**	7	14	7	-50.0
**2021-04**	6	13	6	-53.8
**2021-05**	4	9	5	-44.4
**Relationships - Infidelity**				
**2020-01**	3	8	3	-62.5
**2020-02**	4	9	7	-22.2
**2020-03**	5	10	3	-70.0
**2020-04**	4	9	1	-88.9
**2020-05**	4	10	6	-40.0
**2020-06**	6	12	4	-66.7
**2020-07**	6	13	7	-46.2
**2020-08**	6	13	14	7.7 *
**2020-09**	7	16	6	-62.5
**2020-10**	3	10	6	-40.0
**2020-11**	3	9	9	0.0
**2020-12**	4	12	7	-41.7
**2021-01**	5	12	6	-50.0
**2021-02**	5	10	3	-70.0
**2021-03**	4	9	11	22.2 *
**2021-04**	5	13	4	-69.2
**2021-05**	4	9	6	-33.3
**Relationships - Other relationship distress**				
**2020-01**	9	15	12	-20.0
**2020-02**	9	15	6	-60.0

	Expected number of monthly suicides (No)	95% upper bound of the expected number of monthly suicides (No)	Observed number of suicides (No)	Percent changes (%)
**2020-03**	7	13	8	-38.5
**2020-04**	8	13	4	-69.2
**2020-05**	8	14	9	-35.7
**2020-06**	9	16	5	-68.8
**2020-07**	8	16	9	-43.8
**2020-08**	9	16	13	-18.8
**2020-09**	8	16	12	-25.0
**2020-10**	7	15	17	13.3 *
**2020-11**	8	18	12	-33.3
**2020-12**	9	16	8	-50.0
**2021-01**	9	15	10	-33.3
**2021-02**	9	15	3	-80.0
**2021-03**	5	10	13	30.0 *
**2021-04**	9	16	8	-50.0
**2021-05**	7	13	5	-61.5
**Relationships - Others**				
**2020-01**	1	4	3	-25.0
**2020-02**	2	7	2	-71.4
**2020-03**	2	6	4	-33.3
**2020-04**	2	7	0	-100.0
**2020-05**	1	5	5	0.0
**2020-06**	2	8	2	-75.0
**2020-07**	1	4	2	-50.0
**2020-08**	2	6	5	-16.7
**2020-09**	4	10	5	-50.0
**2020-10**	4	9	6	-33.3
**2020-11**	4	10	5	-50.0
**2020-12**	3	7	1	-85.7

© 2022 Koda M et al. *JAMA Network Open.*

	Expected number of monthly suicides (No)	95% upper bound of the expected number of monthly suicides (No)	Observed number of suicides (No)	Percent changes (%)
**2021-01**	2	5	1	-80.0
**2021-02**	2	8	4	-50.0
**2021-03**	3	7	2	-71.4
**2021-04**	3	8	2	-75.0
**2021-05**	2	7	1	-85.7
**School - Admissions**				
**2020-01**	-	-	1	-
**2020-02**	0	6	2	-66.7
**2020-03**	1	6	1	-83.3
**2020-04**	-	-	0	-
**2020-05**	-	-	0	-
**2020-06**	-	-	2	-
**2020-07**	-	-	0	-
**2020-08**	0	4	3	-25.0
**2020-09**	1	6	0	-100.0
**2020-10**	-	-	1	-
**2020-11**	1	6	1	-83.3
**2020-12**	-	-	1	-
**2021-01**	1	6	2	-66.7
**2021-02**	-	-	0	-
**2021-03**	-	-	1	-
**2021-04**	-	-	0	-
**2021-05**	-	-	0	-
**School - Academic path**				
**2020-01**	2	7	3	-57.1
**2020-02**	3	8	3	-62.5
**2020-03**	2	7	3	-57.1
**2020-04**	2	7	5	-28.6

© 2022 Koda M et al. *JAMA Network Open.*

	Expected number of monthly suicides (No)	95% upper bound of the expected number of monthly suicides (No)	Observed number of suicides (No)	Percent changes (%)
**2020-05**	2	7	3	-57.1
**2020-06**	2	7	5	-28.6
**2020-07**	2	7	4	-42.9
**2020-08**	2	6	6	0.0
**2020-09**	2	8	7	-12.5
**2020-10**	3	8	4	-50.0
**2020-11**	2	5	5	0.0
**2020-12**	3	7	5	-28.6
**2021-01**	3	7	0	-100.0
**2021-02**	2	6	2	-66.7
**2021-03**	3	8	4	-50.0
**2021-04**	4	9	1	-88.9
**2021-05**	4	8	3	-62.5
**School - Academic failure**				
**2020-01**	2	7	3	-57.1
**2020-02**	2	6	2	-66.7
**2020-03**	2	6	0	-100.0
**2020-04**	1	6	2	-66.7
**2020-05**	2	6	2	-66.7
**2020-06**	2	6	4	-33.3
**2020-07**	2	6	3	-50.0
**2020-08**	2	6	5	-16.7
**2020-09**	3	7	2	-71.4
**2020-10**	2	6	2	-66.7
**2020-11**	1	5	2	-60.0
**2020-12**	2	6	4	-33.3
**2021-01**	3	7	4	-42.9
**2021-02**	2	6	4	-33.3

© 2022 Koda M et al. *JAMA Network Open.*

	Expected number of monthly suicides (No)	95% upper bound of the expected number of monthly suicides (No)	Observed number of suicides (No)	Percent changes (%)
**2021-03**	2	6	3	-50.0
**2021-04**	2	5	1	-80.0
**2021-05**	2	6	1	-83.3
**School - Issues with teachers**				
**2020-01**	-	-	0	-
**2020-02**	-	-	0	-
**2020-03**	-	-	0	-
**2020-04**	-	-	0	-
**2020-05**	-	-	0	-
**2020-06**	-	-	0	-
**2020-07**	-	-	1	-
**2020-08**	-	-	0	-
**2020-09**	-	-	1	-
**2020-10**	-	-	0	-
**2020-11**	-	-	0	-
**2020-12**	-	-	0	-
**2021-01**	-	-	1	-
**2021-02**	-	-	0	-
**2021-03**	-	-	0	-
**2021-04**	-	-	0	-
**2021-05**	-	-	0	-
**School - Bullying**				
**2020-01**	-	-	0	-
**2020-02**	-	-	2	-
**2020-03**	-	-	2	-
**2020-04**	-	-	0	-
**2020-05**	-	-	0	-
**2020-06**	-	-	0	-

© 2022 Koda M et al. *JAMA Network Open.*

	Expected number of monthly suicides (No)	95% upper bound of the expected number of monthly suicides (No)	Observed number of suicides (No)	Percent changes (%)
**2020-07**	-	-	0	-
**2020-08**	-	-	0	-
**2020-09**	-	-	0	-
**2020-10**	-	-	0	-
**2020-11**	-	-	0	-
**2020-12**	-	-	0	-
**2021-01**	-	-	0	-
**2021-02**	-	-	0	-
**2021-03**	-	-	2	-
**2021-04**	-	-	1	-
**2021-05**	-	-	1	-
**School - Schoolmate trouble**				
**2020-01**	1	4	1	-75.0
**2020-02**	-	-	1	-
**2020-03**	2	8	2	-75.0
**2020-04**	-	-	0	-
**2020-05**	-	-	1	-
**2020-06**	2	6	4	-33.3
**2020-07**	2	6	2	-66.7
**2020-08**	1	5	8	60.0 *
**2020-09**	3	10	2	-80.0
**2020-10**	1	5	3	-40.0
**2020-11**	2	7	2	-71.4
**2020-12**	1	5	3	-40.0
**2021-01**	2	5	0	-100.0
**2021-02**	1	5	3	-40.0
**2021-03**	2	6	1	-83.3
**2021-04**	1	4	1	-75.0

© 2022 Koda M et al. *JAMA Network Open.*

	Expected number of monthly suicides (No)	95% upper bound of the expected number of monthly suicides (No)	Observed number of suicides (No)	Percent changes (%)
**2021-05**	2	7	3	-57.1
**School - Others**				
**2020-01**	1	6	3	-50.0
**2020-02**	2	6	1	-83.3
**2020-03**	1	4	0	-100.0
**2020-04**	1	5	3	-40.0
**2020-05**	-	-	0	-
**2020-06**	-	-	1	-
**2020-07**	1	6	2	-66.7
**2020-08**	2	6	4	-33.3
**2020-09**	2	7	2	-71.4
**2020-10**	1	6	1	-83.3
**2020-11**	1	4	4	0.0
**2020-12**	2	7	0	-100.0
**2021-01**	1	7	0	-100.0
**2021-02**	1	7	2	-71.4
**2021-03**	-	-	2	-
**2021-04**	1	7	3	-57.1
**2021-05**	2	7	1	-85.7
**Others - Discovery of a crime**				
**2020-01**	-	-	1	-
**2020-02**	-	-	0	-
**2020-03**	1	6	1	-83.3
**2020-04**	1	6	3	-50.0
**2020-05**	2	7	2	-71.4
**2020-06**	1	6	5	-16.7
**2020-07**	2	9	3	-66.7
**2020-08**	1	7	2	-71.4

© 2022 Koda M et al. *JAMA Network Open.*

	Expected number of monthly suicides (No)	95% upper bound of the expected number of monthly suicides (No)	Observed number of suicides (No)	Percent changes (%)
**2020-09**	1	6	3	-50.0
**2020-10**	1	6	1	-83.3
**2020-11**	1	5	4	-20.0
**2020-12**	2	7	2	-71.4
**2021-01**	2	5	2	-60.0
**2021-02**	1	4	2	-50.0
**2021-03**	2	5	1	-80.0
**2021-04**	2	5	1	-80.0
**2021-05**	3	7	2	-71.4
**Others - Victim of a crime**				
**2020-01**	-	-	1	-
**2020-02**	-	-	1	-
**2020-03**	-	-	1	-
**2020-04**	-	-	0	-
**2020-05**	-	-	1	-
**2020-06**	-	-	0	-
**2020-07**	-	-	1	-
**2020-08**	0	5	3	-40.0
**2020-09**	-	-	0	-
**2020-10**	-	-	1	-
**2020-11**	-	-	0	-
**2020-12**	-	-	0	-
**2021-01**	-	-	0	-
**2021-02**	-	-	0	-
**2021-03**	-	-	0	-
**2021-04**	-	-	0	-
**2021-05**	-	-	0	-
**Others - Copycat suicide**				

© 2022 Koda M et al. *JAMA Network Open.*

	Expected number of monthly suicides (No)	95% upper bound of the expected number of monthly suicides (No)	Observed number of suicides (No)	Percent changes (%)
**2020-01**	2	5	5	0.0
**2020-02**	2	6	2	-66.7
**2020-03**	2	5	5	0.0
**2020-04**	3	6	0	-100.0
**2020-05**	1	4	3	-25.0
**2020-06**	2	5	1	-80.0
**2020-07**	2	5	1	-80.0
**2020-08**	2	6	4	-33.3
**2020-09**	3	8	9	12.5 *
**2020-10**	4	12	10	-16.7
**2020-11**	4	13	5	-61.5
**2020-12**	4	10	2	-80.0
**2021-01**	3	8	4	-50.0
**2021-02**	4	12	4	-66.7
**2021-03**	3	8	5	-37.5
**2021-04**	3	8	0	-100.0
**2021-05**	1	6	5	-16.7
**Others - Loneliness**				
**2020-01**	8	14	11	-21.4
**2020-02**	11	18	10	-44.4
**2020-03**	11	18	10	-44.4
**2020-04**	10	17	10	-41.2
**2020-05**	10	17	8	-52.9
**2020-06**	9	16	15	-6.2
**2020-07**	13	20	19	-5.0
**2020-08**	13	23	12	-47.8
**2020-09**	11	19	13	-31.6
**2020-10**	11	18	18	0.0

© 2022 Koda M et al. *JAMA Network Open.*

	Expected number of monthly suicides (No)	95% upper bound of the expected number of monthly suicides (No)	Observed number of suicides (No)	Percent changes (%)
**2020-11**	11	20	20	0.0
**2020-12**	13	23	11	-52.2
**2021-01**	9	15	13	-13.3
**2021-02**	11	18	11	-38.9
**2021-03**	10	17	12	-29.4
**2021-04**	10	16	14	-12.5
**2021-05**	12	19	16	-15.8
**Others - Neighborhood trouble**				
**2020-01**	1	5	1	-80.0
**2020-02**	1	4	1	-75.0
**2020-03**	-	-	1	-
**2020-04**	2	5	3	-40.0
**2020-05**	2	6	2	-66.7
**2020-06**	2	6	0	-100.0
**2020-07**	1	4	4	0.0
**2020-08**	2	6	1	-83.3
**2020-09**	2	5	2	-60.0
**2020-10**	2	6	1	-83.3
**2020-11**	2	5	3	-40.0
**2020-12**	2	6	1	-83.3
**2021-01**	1	4	0	-100.0
**2021-02**	1	4	2	-50.0
**2021-03**	2	6	2	-66.7
**2021-04**	2	6	1	-83.3
**2021-05**	2	5	1	-80.0
**Others - Others**				
**2020-01**	10	19	8	-57.9
**2020-02**	10	17	5	-70.6

© 2022 Koda M et al. *JAMA Network Open.*

	Expected number of monthly suicides (No)	95% upper bound of the expected number of monthly suicides (No)	Observed number of suicides (No)	Percent changes (%)
**2020-03**	8	14	9	-35.7
**2020-04**	11	17	5	-70.6
**2020-05**	10	17	10	-41.2
**2020-06**	10	17	14	-17.6
**2020-07**	9	16	15	-6.2
**2020-08**	9	18	15	-16.7
**2020-09**	10	17	11	-35.3
**2020-10**	10	16	24	50.0 *
**2020-11**	12	24	13	-45.8
**2020-12**	9	15	16	6.7 *
**2021-01**	9	18	9	-50.0
**2021-02**	8	14	7	-50.0
**2021-03**	7	12	9	-25.0
**2021-04**	8	15	12	-20.0
**2021-05**	10	17	13	-23.5

Percent change was defined as the difference between the observed number of suicides for a month and the 95% upper bound of the expected number of suicides for that month divided by the threshold. * Indicates a month with the observed number of suicides exceeding the 95% upper bound of the expected number of suicides for that month. - Indicates a month for which we were unable to calculate the percent owing to a small sample.
